# TraDIS-Xpress: a high-resolution whole-genome assay identifies novel mechanisms of triclosan action and resistance

**DOI:** 10.1101/gr.254391.119

**Published:** 2020-02

**Authors:** Muhammad Yasir, A. Keith Turner, Sarah Bastkowski, David Baker, Andrew J. Page, Andrea Telatin, Minh-Duy Phan, Leigh Monahan, George M. Savva, Aaron Darling, Mark A. Webber, Ian G. Charles

**Affiliations:** 1 Quadram Institute Bioscience, Norwich Research Park, Norwich, NR4 7UQ, United Kingdom;; 2 School of Chemistry and Molecular Biosciences, The University of Queensland, St. Lucia 4072, Queensland, Australia;; 3 Faculty of Science, University of Technology Sydney, New South Wales 2007, Australia;; 4 University of East Anglia, Norwich Research Park, Norwich, NR4 7TJ, United Kingdom

## Abstract

Understanding the genetic basis for a phenotype is a central goal in biological research. Much has been learnt about bacterial genomes by creating large mutant libraries and looking for conditionally important genes. However, current genome-wide methods are largely unable to assay essential genes which are not amenable to disruption. To overcome this limitation, we developed a new version of “TraDIS” (transposon directed insertion-site sequencing) that we term “TraDIS-Xpress” that combines an inducible promoter into the transposon cassette. This allows controlled overexpression and repression of all genes owing to saturation of inserts adjacent to all open reading frames as well as conventional inactivation. We applied TraDIS-Xpress to identify responses to the biocide triclosan across a range of concentrations. Triclosan is endemic in modern life, but there is uncertainty about its mode of action with a concentration-dependent switch from bacteriostatic to bactericidal action unexplained. Our results show a concentration-dependent response to triclosan with different genes important in survival between static and cidal exposures. These genes include those previously reported to have a role in triclosan resistance as well as a new set of genes, including essential genes. Novel genes identified as being sensitive to triclosan exposure include those involved in barrier function, small molecule uptake, and integrity of transcription and translation. We anticipate the approach we show here, by allowing comparisons across multiple experimental conditions of TraDIS data, and including essential genes, will be a starting point for future work examining how different drug conditions impact bacterial survival mechanisms.

Triclosan is a broad-spectrum antimicrobial that was first developed in the 1960s. Since then, it has been used extensively in a wide range of products for clinical, veterinary, and domestic use (Yazdankhah et al. 2006), such as hand soaps, mouthwashes, toothpaste, shampoos, and cosmetics, as well as being incorporated into plastics. Triclosan acts by inhibiting type II fatty acid biosynthesis and is the archetypal inhibitor of this essential pathway in bacteria. Over the years, other compounds that inhibit enzymes in this pathway have progressed to different stages of clinical development as novel antibiotics, but triclosan is the only one in widespread domestic use. Triclosan specifically inhibits the enzyme FabI; an enoyl-acyl carrier protein (ACP) reductase conserved across bacteria; hence, triclosan has a correspondingly broad spectrum of activity against most bacteria (Russell 2004).

The use of triclosan has proliferated to such an extent that it is readily identified in most aquatic environments and is detectable in the urine and blood plasma of most people in the developed world, where use has been extensive in personal care products, particularly toothpastes (Toms et al. 2011; Mathews et al. 2014; Savage et al. 2014). A U.S. study found ∼10% of adults had levels of triclosan in their urine above the minimum inhibitory concentration required to inhibit most bacteria (Calafat et al. 2008).

There has been pressure to ban triclosan use owing to concerns about its potential impact as a hormone-disrupting agent and evidence that exposure to triclosan can select for mutants cross-resistant to antibiotics (Webber et al. 2013; Li et al. 2019; Westfall et al. 2019). Evidence from mouse studies has suggested that triclosan exposure can alter the composition of the gut microbiota and may induce inflammation and promote colonic cancers (Yang et al. 2018). The impact of triclosan in urban (built) environments, where it is now a common contaminant in developed countries (Fahimipour et al. 2018), has recently been investigated. Correlations between triclosan concentrations and organisms present in dust strongly suggest the selection of drug-resistant bacterial communities in the presence of triclosan (Fahimipour et al. 2018).

Although the primary target of triclosan has been established as FabI, a full understanding of triclosan mechanisms of action has remained elusive. It has been shown that triclosan exerts a bacteriostatic effect at low concentrations (McMurry et al. 1998b) but, at higher concentrations, is bactericidal (McMurry et al. 1998b). Triclosan resistance also appears multifactorial; highly resistant mutants typically have substitutions within FabI that reduce binding efficiency of triclosan, but multidrug efflux pumps, barrier function, changes to *fabI* expression, core metabolic pathways and sigma factor–controlled stress responses have all been implicated as being involved in triclosan resistance (McMurry et al. 1998a; Webber et al. 2008a,b; Bailey et al. 2009; Gantzhorn et al. 2015). A specific link between resistance to fluoroquinolone antibiotics and triclosan, mediated by altered stress responses present in *gyrA* mutants, has also recently been described (Webber et al. 2013).

Given renewed interest in the impact of triclosan on population health and the microbiome, we felt it was timely to systematically investigate the mechanisms of action and resistance of triclosan across a range spanning bacteriostatic and bactericidal concentrations. This will help understanding of the potential impacts and risks of exposure to triclosan under different conditions as well as informing fatty acid inhibitor development programs and providing further insight into the impacts of triclosan on the microbiome.

Previous systems biology approaches to investigate bacterial responses to triclosan have focused on nucleotide sequencing the genomes of mutants or on transcriptomic and proteomic analysis that have tended to use a small number of concentrations (Webber et al. 2008a; Bailey et al. 2009). These studies have been informative, although each is limited to providing a snapshot of genes potentially involved in triclosan susceptibility at one condition.

Here we applied a modified version of “TraDIS” (transposon directed insertion-site sequencing) (Langridge et al. 2009; Barquist et al. 2016) to identify genes involved in sensitivity to triclosan over a wide range of concentrations. TraDIS, or other analogous approaches relying in high-throughput sequencing of transposon mutant libraries such as Tn-seq or HITS (van Opijnen and Camilli 2013), combines production of a large, saturating transposon mutant library with sequencing of all transposon–chromosome insertion junction sites in parallel. Differences in insertion sites when grown in different conditions identify genes involved in survival of any given test condition. However, traditional TraDIS protocols have been limited by being unable to assay essential genes that do not tolerate any insertions within them, as well as by being costly and generating large volumes of data, which are difficult to analyze. Our new methodology uses a transposon with an outward-directed inducible promoter, allowing the impact of gene overexpression and gene silencing of every gene in a candidate organism to be assayed (in addition to insertional inactivation), and we refer to this approach as TraDIS-Xpress. By using TraDIS-Xpress, we analyzed the response of *Escherichia coli* to triclosan at eight concentrations spanning a 125-fold range. We also used a refined analysis pipeline we have developed (AlbaTraDIS) to identify and compare changes in insertion site abundance from multiple exposure conditions, as well as to automatically predict impacts of insertions on gene function and expression.

By using this approach, we show distinct concentration-dependent responses to triclosan and identify novel genes involved in triclosan susceptibility, including the identification of potential routes of entry into the cell. We were also able to systematically document different impacts on distinct sets of genes between exposure to bacteriostatic and bactericidal concentrations of the drug for the first time. The ability to regulate expression of all the genes of a target organism in one massively parallel experiment in a controlled manner promises to be a hugely powerful strategy that has the potential to uncover new genotype–phenotype relationships.

## Results

### Creation of a high-density transposon insert library in *E. coli* strain BW25113

*E. coli* BW25113 was transformed with a bespoke Tn5-based transposon containing an outward facing *tac* promoter. To confirm that a suitable number of unique transposon inserts had been harvested and that the modified TraDIS-Xpress sequencing strategy worked, DNA was extracted from the transposon mutant library pool and was processed for TraDIS sequencing (Supplemental Fig. S1). In this experiment, 5.1 million TraDIS reads were mapped to the BW25113 reference genome at 813,873 unique sites, giving an average of one insertion site every 6 bp (Supplemental Fig. S2). Analysis of the insertion sites revealed an expected lack of inserts in genes previously reported as essential (Goodall et al. 2018). These data confirmed that a high-density mutant library covering the genome had been created and that the modified TraDIS sequencing method worked as expected. We also assessed the reproducibility of experiments by comparing data from two separate cultures seeded from the library by sequencing transposon insertion sites in control conditions and in the presence of three concentrations of triclosan (Supplemental Fig. S2). These data showed excellent correlation between the replicates. Finally, 48 individual transposon mutants were randomly selected and whole-genome-sequenced to check insertion sites. Bioinformatic analysis of these mutants confirmed that each mutant contained only one copy of the transposon inserted in the genome at unique positions, thus supporting the assertion that the transposon mutant library pool consists largely of mutants with single transposon insertions.

A key advantage of TraDIS-Xpress is the use of an inducible promoter that allows expression changes to be inferred and gives the ability to score essential genes for involvement in a phenotype. To assess the utility of promoter induction, we determined the number of loci reported as involved in triclosan susceptibility in each condition in the presence and absence of induction (Supplemental Fig. S3). These data revealed additional information was retrieved for seven of the eight test conditions, with an average of 21% more targets being identified when induction of the outward facing promoter was applied.

### TraDIS-Xpress is a powerful tool that identifies multiple genes involved in triclosan sensitivity

**Figure 1.**
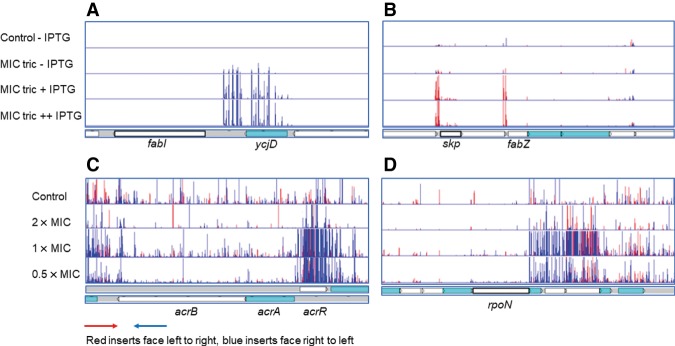
Validation of TraDIS-Xpress. Identification of known targets using the TraDIS-Xpress approach incorporating an inducible outward-facing promoter that identifies the impact of both essential and nonessential genes on survival and growth. (*A*) Genetic map of the relative gene positions is shown at the *bottom* of the panel. *Above* this, each row of vertical red or blue lines (plotted with red behind) indicates the position of mapped reads, and the height of the bar represents the relative number of reads mapped. Red indicates transposon insertions wherein the transposon-encoded kanamycin-resistance gene is oriented 5′ to 3′ left to right, and blue indicates the opposite right-to-left orientation. (*A*,*B*) Data split with induced and uninduced libraries shown at a single concentration of triclosan; this makes the impact of induction obvious. (*C*,*D*) Results from different drug concentrations (all with induction). (*A*) Inserts predicted to result in up-regulation of *fabI* in the presence of triclosan. (*B*) Mutants up-regulating *skp*, *lpxD*, *fabZ*, *lpxA*, and *lpxB*. (*C*) Mutants inactivating *acrR* and up-regulating *acrAB* being enriched by triclosan. (*D*) Mutants positioned antisense to *rpoN* being selected in the presence of triclosan. The *top* row in each plot shows untreated control cultures; the three rows *below* are for cultures grown in the presence of 2×, 1×, and 0.5× the MIC of triclosan (with IPTG induction for the outward facing promoter).

A total of 66 independent TraDIS experiments were completed across a 125-fold concentration range of triclosan. Analysis of the differential abundance of insertion sites in the presence of triclosan identified previously reported mechanisms of triclosan resistance as highly significant targets. FabI is the product of the essential gene *fabI*, which cannot be inactivated by transposon insertion. Our data (Fig. 1) revealed a highly significant enrichment of transposon inserts upstream of *fabI* in triclosan-treated experiments (these were present upon the addition of IPTG but not in uninduced conditions) with a very strong bias for transposon insertions oriented such that the outward directed promoter will up-regulate transcription of *fabI*. This confirmed that we were able to identify known mechanisms of triclosan resistance, and shows that TraDIS-Xpress can readily identify the contribution of an essential gene to survival following exposure to a target drug.

Comparison of results from libraries exposed to triclosan in the presence of IPTG with those without addition of inducer clearly showed the enrichment of insert sites upstream of and downstream from genes in the presence of the inducer (Fig. 1). This included various essential genes in which overexpression or repression owing to antisense insertion sites was enriched by triclosan exposure only in the presence of inducer. These data confirmed that TraDIS-Xpress was able to efficiently assay the roles of essential genes in triclosan sensitivity and that there was not significant “leakiness” of the outward facing promoter without induction.

Multidrug efflux pumps and outer membrane porins have also been shown previously to influence triclosan sensitivity, including the multidrug efflux system AcrAB. Mutants with inserts within the genes encoding the structural components of this pump were depleted in the presence of triclosan concentrations above the MIC (Fig. 1), whereas inserts within the local repressor of the system, *acrR*, were enriched. Similarly, mutants within outer membrane porin, *ompF*, were enriched in the presence of triclosan. Expression of *acrAB* and *ompF* are controlled by global stress response systems, *marRA* and *soxRS*; inserts within *marR* and *soxR* (both of which result in derepression of the cognate transcriptional activator genes, which then promote pump expression) were significantly enriched upon triclosan exposure. Inactivation of sigma factors has also been associated previously with triclosan resistance (Gantzhorn et al. 2015); in our data, there was significant enrichment of inserts immediately downstream from *rpoN* (Fig. 1) but in an antisense orientation in the presence of triclosan, showing that inducible promoters can also identify genes in which repression is beneficial following exposure to a given stress. Reducing expression of *rpoN* is likely to alter the cell response to stress, although the direct impact of individual effectors on triclosan sensitivity is unclear.

To assess the validity of predictions made by TraDIS-Xpress, we selected 78 mutants from the Keio collection in which altered sensitivity to triclosan was predicted, three randomly selected mutants from the collection, and a set of other importers. We modelled growth and compared each mutant to the parent and assessed how the fitness score from AlbaTraDIS related to growth ability in the presence of triclosan. Supplemental Figure S4 shows how each mutation affected the ratio of growth with no antimicrobial with growth in 0.125 mg/L triclosan, compared to the same ratio in BW25113. This shows that, on average, mutants followed the prediction by TraDIS-Xpress, with the majority showing less sensitivity to triclosan than the wild type and a smaller set (including the expected efflux mutants) showing increased sensitivity. The random mutants not indicated by TraDIS did not show any detectable change in their susceptibility. Comparisons of the AUC values showed most mutants followed the predicted impacts; 92% of the mutants showed a change in AUC >20% ± the parent strain in line with the prediction made by TraDIS-Xpress, although the statistical power of this comparison is weak for any individual mutant. Supplemental Figure S5 shows the relationship between TraDIS-Xpress fitness scores with these growth data (both in the presence of 0.125 mg/L of triclosan), although those mutants with the clearest positive and negative signals from TraDIS-Xpress have clear corresponding effects seen in growth experiments. There is, however, little evidence of a general correlation between the strength of a TraDIS-Xpress log fold change and corresponding growth ability.

### Concentration-dependent results of exposure to triclosan are clearly revealed

**Figure 2.**
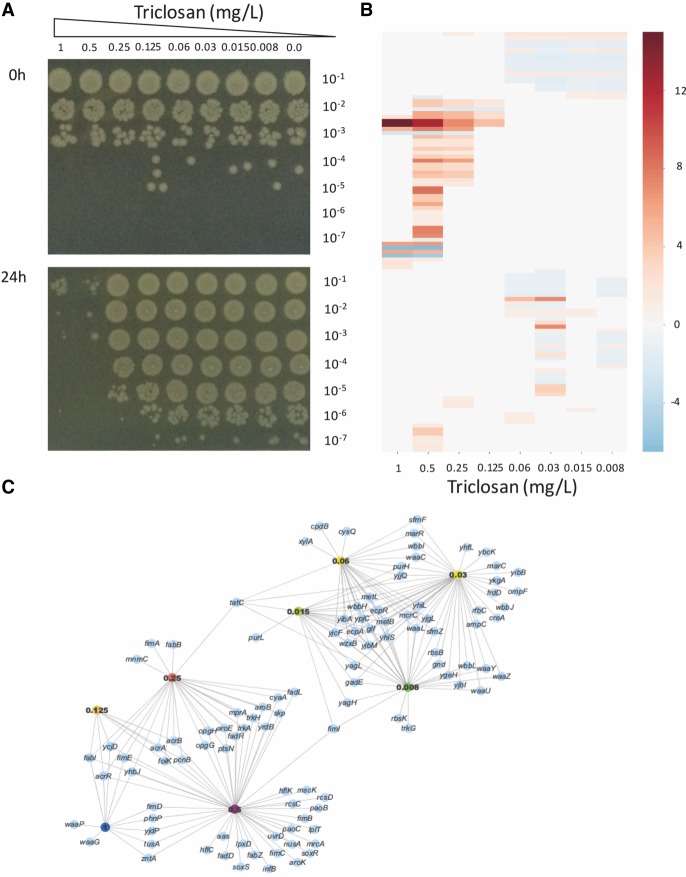
Dose-dependent activity of triclosan. (*A*) Viability of BW25113 from LB broth cultures supplemented with different concentrations of triclosan, immediately after inoculation (*top*) and after incubation for 24 h (*bottom*). Samples from the cultures were serially diluted 10-fold, and 5 µL of all dilutions was spotted onto LB agar and incubated. The level of dilution (from 10^−1^ to 10^−7^) is shown on the *right* side of the photographs, and the concentrations of triclosan in the cultures are shown along the *top*. The results show a bactericidal effect >0.5 mg/L; triclosan at 0.25 mg/L caused a 10-fold reduction in growth over the 24-h period, but at ≤0.125 mg/L, there was no growth inhibition. (*B*) Heat map highlighting differences in reads mapped to genes at the different triclosan concentrations shown along the *bottom* of the chart. Each row represents a gene, and genes are ordered from *top* to *bottom*. The intensity scale reflecting the size of difference is shown on the *right* of the map. (*C*) Network produced by the AlbaTraDIS software illustrating the genes identified as important at different concentrations of triclosan. Nodes represent genes (blue) or triclosan concentrations (indicated by colored nodes); edges show links between conditions and genes. The similarities of responses between sub-MIC and supra-MIC exposures are clearly visible.

Although the well-established mechanisms of triclosan resistance were identified in our data, there were also many genes identified that have not previously been implicated in triclosan resistance. The genes identified were concentration dependent, and exposure to different concentrations of triclosan resulted in a range of impacts on cellular viability (Fig. 2).

Similarly, distinct patterns of transposon mutants were revealed across the range of drug exposures. Two major groups of responses were evident from analysis of the data; one block of genes was selected by exposure to concentrations of triclosan at the MIC and below, and another block was selected by exposure to concentrations above the MIC (Table 1; Fig. 2; Supplemental Table 1). Genes involved in fimbriae biosynthesis (*fimABCDEI*) were selected across a wide range of concentrations.

**Table 1.**
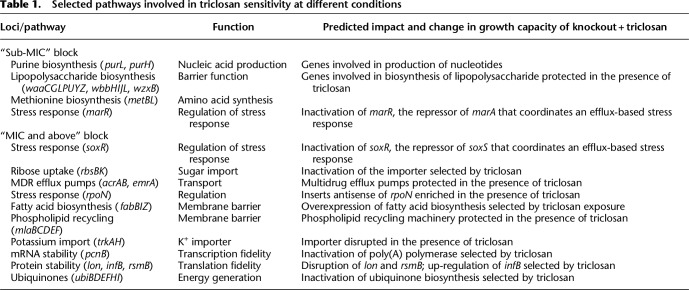
Selected pathways involved in triclosan sensitivity at different conditions

The “MIC and below” block was characterized by the protection of genes encoding lipopolysaccharide biosynthesis, purine biosynthesis, and methionine biosynthesis. Inactivation of *marR*, the repressor of the global stress response regulator, *marA*, was also selected in this block.

The “above MIC” block included mutants that result in derepression of the global stress regulator, *soxS*, which (as with *marA*) controls expression of *acrAB* and repression of the major porin, *ompF*. Together, these will result in reduced permeability of the cell to triclosan (Blair et al. 2014). This block also identified protection of the structural components of the AcrAB multidrug efflux system and overexpression of another efflux system, EmrAB. In addition, inactivation of the ribose uptake system RbsACB and potassium uptake transporter TrkAH was also predicted to be protective of exposure to triclosan. Other loci identified as contributing to survival at the highest concentrations of triclosan included fatty acid biosynthetic genes (including *fabI*), the phospholipid recycling pathway (*mlaBCDEF*), ubiquitins (*ubiDEFGHL*), and *rpoN*. Additionally, a set of genes all involved in the stability of mRNA, proteins, and translation initiation (*pcnB*, *infB*, *lon*, and *rsmB*) was also identified as contributing to survival at high triclosan concentrations (Table 1).

### Triclosan sensitivity is impacted by ribose and potassium import systems

**Figure 3.**
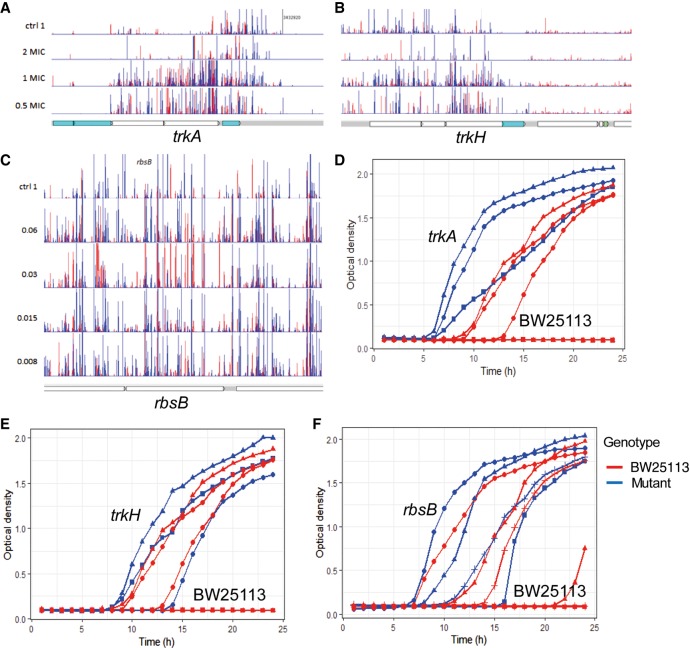
Novel importers contributing to triclosan sensitivity. (*A*,*B*) Insert patterns indicating inactivation of *trkAH* is beneficial for survival in the presence of triclosan. (*C*) Increase in insertions within the first part of *rbsB* in triclosan-exposed libraries. (*D*–*F*) Growth curves for BW25113 and isogenic mutants grown in LB broth supplemented with 0.125 mg/L of triclosan. Mutants were replicated on multiple plates. Each line indicates the average from an individual plate; independent plates are represented by symbols (triangles, circles, or squares; allowing comparison between the mutant and parent from each individual plate). Red lines indicate BW25113; blue lines, mutants. The format of the genetic maps is as described in Figure 1.

Although triclosan has a validated intracellular target in FabI, it is currently unclear how it crosses the cytoplasmic membrane. TraDIS data identified inactivation of *rbsB*, the periplasmic ribose binding domain of the RbsABC ribose importer and *trkHA*, components of a potassium importer as being beneficial to survival in the presence of triclosan. Defined *rbsB*, *trkH*, or *trkA* mutants from the Keio mutant collection showed significantly (*P* < 0.01) better growth compared with BW25113 in the presence of 0.125 mg/L of triclosan, although none were able to grow at 0.5 mg/L (Fig. 3). These data support the hypothesis that these transporters are involved in triclosan sensitivity.

### Genes maintaining transcript and protein stability are involved in triclosan sensitivity

**Figure 4.**
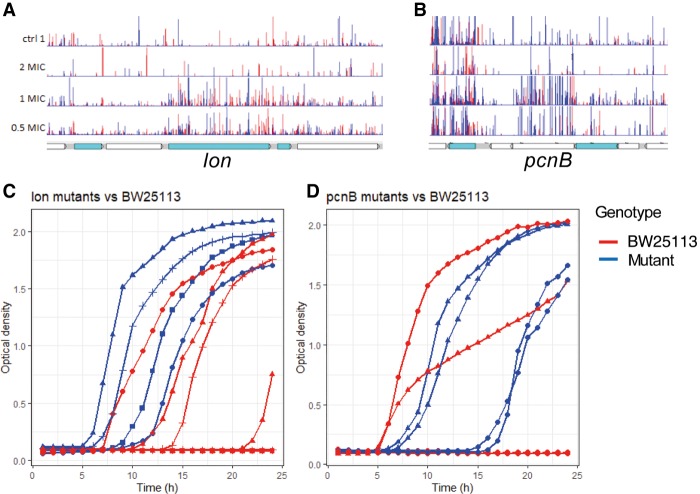
Impact of *lon* and *pcnB* on triclosan sensitivity. (*A*,*B*) Insert patterns indicating inactivation of *lon* and *pcnB* is beneficial for survival in the presence of triclosan. The format of these genetic maps is as described in Figure 1. (*C*,*D*) Growth curves for BW25113 and isogenic mutants grown in LB broth supplemented with 0.125 mg/L of triclosan. Mutants were replicated on multiple plates. Each line indicates the average from an individual plate; independent plates are represented by symbols (triangles, circles, or squares; allowing comparison between the mutant and parent from each individual plate). Red lines indicate BW25113; blue lines, mutants.

The mechanism by which triclosan exerts a bactericidal effect at high concentrations is not understood. TraDIS-Xpress identified a set of genes that are involved in mRNA stability, initiation of translation, and protein stability as being relevant to survival in the presence of high concentrations of triclosan. These included *pcnB*, which adds poly(A) tails to newly synthesized mRNA transcripts, which target their digestion; inactivation of *pcnB* gave a selective advantage during growth in triclosan (Fig. 4). TraDIS-Xpress also identified a selective advantage in triclosan for mutants with insertions immediately upstream of both *infB* and *rsmB*, which code for a translation initiation factor and a methyltransferase acting to stabilize binding of initiator tRNAs to the ribosome preinitiation complex, respectively. Finally, mutants with inactivation of the Lon protease were found to survive better in the presence of triclosan (Fig. 4). Lon is a general protease (previously implicated in processing of AcrB). These observations suggest that exposure to higher triclosan concentrations impairs mRNA transcript stability and protein translation; thus, mutations that result in increased stability of mRNA and proteins provide a selective advantage.

### Modulating expression of triclosan sensitivity genes confirmed predictions about the impacts of specific genes on survival in the presence of triclosan

**Figure 5.**
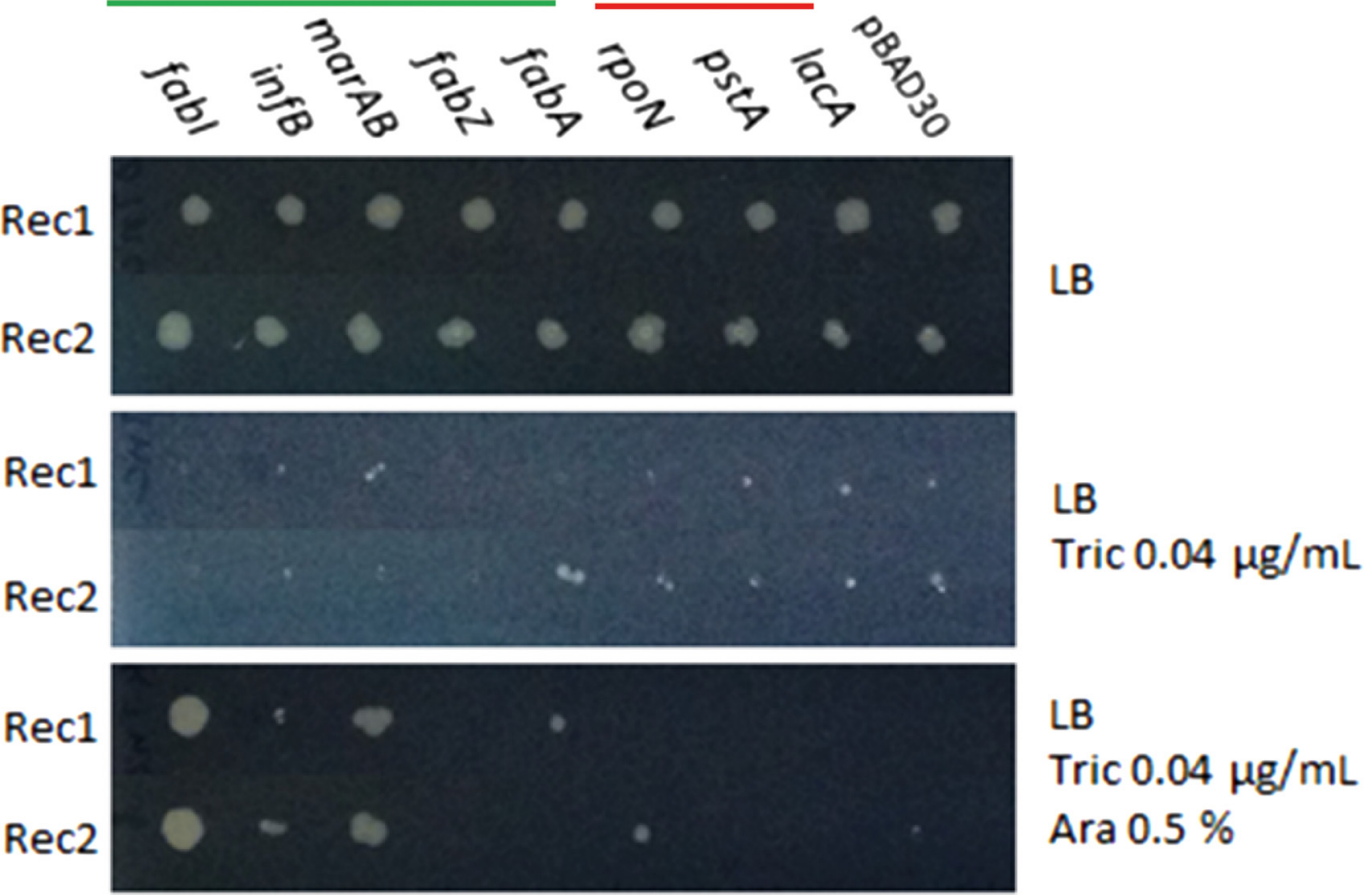
Altering expression of targets impacts triclosan sensitivity. The coding sequences of selected genes were cloned into the pBAD30 expression vector under transcriptional control of the arabinose-inducible promoter. This was to modify their expression and test the predicted impact on triclosan sensitivity. Genes *fabI*, *infB*, *marAB*, and *fabZ* were cloned in the forward orientation, allowing their up-regulation after induction (under the green bar). In contrast, *fabA*, *rpoN*, *pstA*, and *lacA* were cloned in the reverse direction, allowing inducible production of an antisense transcript and consequent repression of expression (under the red bar). The *lacA* and pBAD30 vector alone constructs were included as negative controls. Duplicate recombinants (Rec1, Rec2) for each gene were tested by spotting cultures onto LB-agar (*top*), LB agar supplemented with 0.04 µg/mL triclosan (*middle*), or LB agar supplemented with 0.04 µg/mL triclosan and 0.5% arabinose. All recombinants grew without selection, and none grew in the presence of triclosan without arabinose. With triclosan and arabinose induction, the *fabI*, *infB*, and *marAB* recombinants consistently grew better than the controls.

A key advantage of using promoters in transposon mutant libraries is the ability to assay essential genes that cannot be inactivated for roles in stress responses. TraDIS-Xpress identified numerous genes in which the pattern of transposon inserts suggested expression changes were altering triclosan sensitivity. For some of these genes, a growth advantage with triclosan was predicted owing to transposon insertions downstream from the gene and to the production of an antisense transcript that will interfere with translation of mRNA. To verify these predictions, a selection of these genes was chosen for artificial up- or down-regulation. A series of constructs were made in the pBAD gene expression vector (Fig. 5), with some inserted in reverse orientation to mimic observations from the TraDIS-Xpress data. None of the *E. coli* derivatives harboring these plasmid constructs showed any change in growth rate on triclosan-free media. After induction with arabinose, constructs overexpressing *fabI*, *infB*, and *marA* showed consistent “rescue” of the ability to grow in the presence of 0.5 mg/L on agar. Constructs overexpressing *fabA*, or antisense to *rpoN* and *pstA*, showed rescue of growth ability in the presence of triclosan in broth (consistently showing an ability to grow in concentrations of triclosan one or two dilutions higher than the parent), although this was not reproduced when grown on agar. The original TraDIS-Xpress experiments were completed in broth, and these genes may have inherently different baseline levels of expression when cells are grown on a solid medium, which may explain this mismatch. Artificial overexpression of *fabZ* appeared to be consistently lethal. Vector-only controls or a clone containing *lacA* (chosen as a random gene not implicated in triclosan sensitivity) showed no phenotype, as expected (Fig. 5).

## Discussion

The use of large transposon libraries and massively parallel sequencing to assay the fitness of large numbers of mutants has proved powerful in defining the essential set of genes needed for any bacteria to grow in any specific condition. For example, a recent report refined the *E. coli* essential gene list in common laboratory conditions and identified essential regions within genes (Goodall et al. 2018). Studies using the original TraDIS methodology are, however, limited by not being able to assay essential genes and by the time taken to make mutant libraries and sequencing costs, which are high as “diversity” samples often need to be added to the flow cell if using an Illumina sequencing instrument, reducing the amount of data that can be produced from the experimental samples per run. Improved workflows included the use of outward-facing promoters of different strengths have helped to address this shortcoming, and the cost per typical TraDIS-Xpress experiment has now been reduced significantly compared with a corresponding experiment with the original TraDIS workflow. Cost savings result from use of conjugative plasmids to deliver transposons in library construction rather than multiple rounds of laborious electroporation, sequencing primers that are included making the workflow compatible with standard Illumina library preparation and sequencing protocols and improved bioinformatics. All these reduce hands-on time significantly, and our current workflows are approximately threefold cheaper in consumables costs and approximately fivefold faster than conventional protocols. Supplemental Table 2 summarizes time and cost savings with TraDIS Xpress compared with the original TraDIS approach.

Other methods to assay the role of all known genes within a bacterium in a phenotype of interest have been attempted, including the recent application of CRISPRi (Lee et al. 2019) in which genes are silenced, although this requires prior knowledge of genes to target and cannot assess the impact of gene overexpression on a phenotype, or mutation, of intergenic regions. Our method also provides much greater resolution; we introduced an average of approximately 125 inserts into each gene, allowing the essentiality of domains encoded by parts of a gene to be tested rather than single knockdown of a gene. We also assay all the noncoding parts of the genome in this approach.

Here we have incorporated inducible control to an outward-facing promoter integrated into the transposon sequence, providing improved control of the expression levels of adjacent genes. This approach has also improved “signal to noise” and allowed comparisons of induced and uninduced conditions to identify genes in which expression changes contribute to conditional survival. The use of a single, inducible promoter rather than a suite of transposons with promoters of different strengths helps avoid loss of density owing to only a subset of each library carrying each promoter. It also avoids any site-specific insertional biases that may arise from the different sequences of a suite of different transposons. Additionally, it has proved a suitable approach to identify where “knockdown” of expression of a gene can influence survival, which we show has identified previously unknown targets. The technology thus enables rapid and simultaneous genome-wide genotype to phenotype association determinations.

In addition, we have developed the “AlbaTraDIS” analysis software suite, which performs the automated identification of significant changes in mapped sequence reads that reflect the changes in the different mutant numbers between growth conditions. “AlbaTraDIS” identifies significant insertion sites in a context- and gene-agnostic manner and then identifies those inserts likely to inactivate genes or alter their expression. Comparisons across diverse conditions allows commonalities and differences in responses to a stress to be easily identified, allowing analysis and integration of large numbers of data sets in an efficient manner.

**Figure 6.**
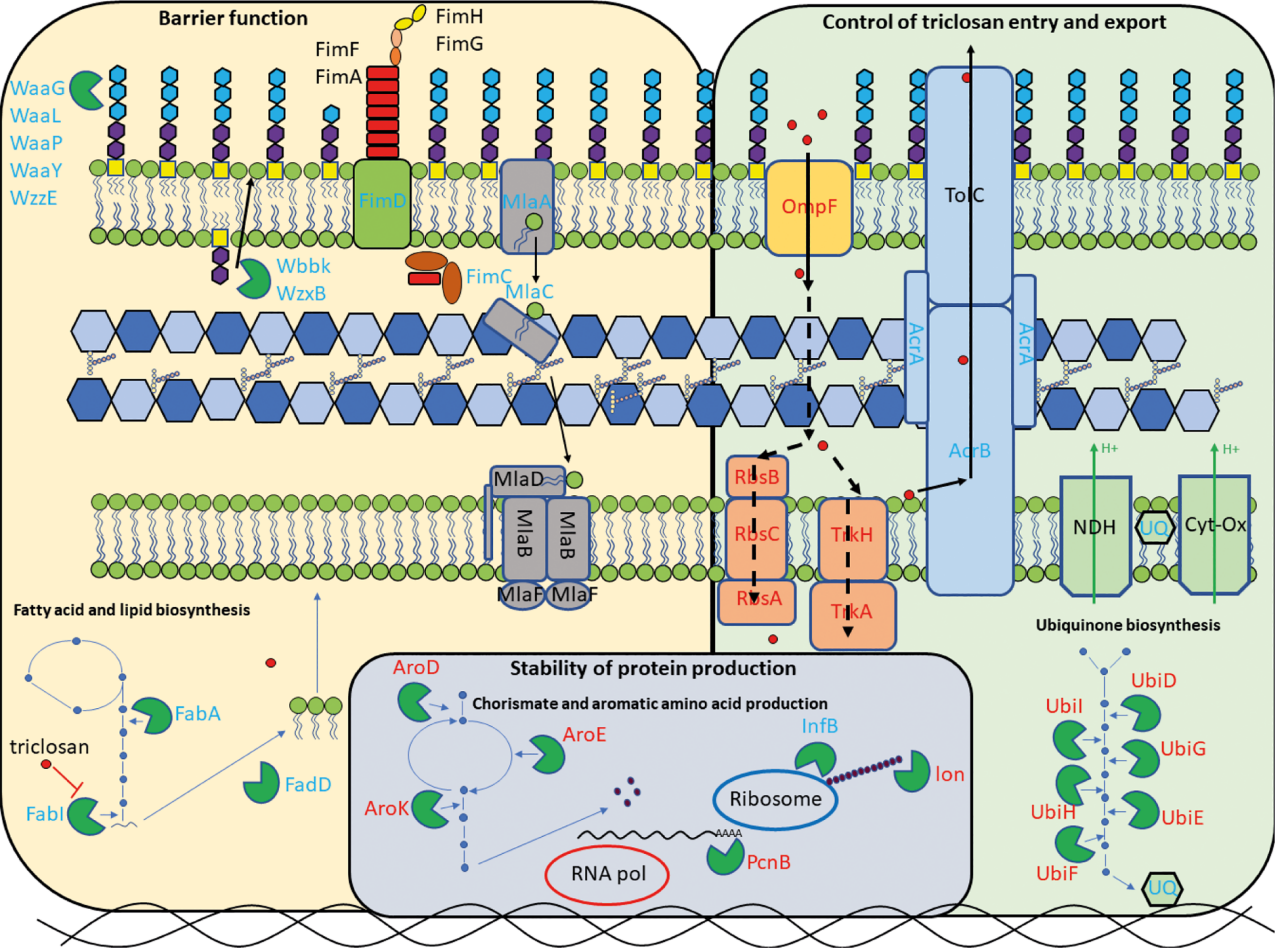
Overview of the major cellular pathways revealed as being involved in triclosan sensitivity by TraDIS-Xpress. Proteins with blue name labels appear beneficial to survival in the presence of triclosan; those with red name labels contribute to triclosan sensitivity. Dotted lines indicate potential routes of triclosan movement across the inner membrane.

We have used TraDIS-Xpress to study mechanisms of resistance to triclosan across a wide range of drug concentrations. The data revealed the known mechanisms of triclosan resistance and allowed us to identify essential genes involved in triclosan sensitivity (including the known primary target) based on expression changes. These experiments validate the use of outward facing promoters in the TraDIS-Xpress method and show that the AlbaTraDIS software suite can identify inserts efficiently and predict impacts on gene expression in an automated manner. Having an automated analysis pipeline allows multiple conditions to be analyzed in conjunction and the relationships between different stress conditions to be inferred (Fig. 2). By using this approach, we were able for the first time to differentiate the differential impacts of triclosan exposure at high and low concentrations. These data are summarized in Figure 6, showing an integrated diagram highlighting the fundamental distinct mechanisms of triclosan action and resistance revealed by TraDIS-Xpress. Previous work with other antibiotics and *E. coli* has identified hermetic effects in which different doses impacted transcription of a set of genes differently (Mathieu et al. 2016). Our data also find clear differences in responses between different exposure conditions, although at a greater resolution and with higher sensitivity. Analysis of the comparison between “strength” of a TraDIS-Xpress score and resulting impact in growth kinetics did not show a strong correlation. The experiment was sensitive in identifying conditionally important genes, but the degree of effect was harder to predict with accuracy. This may not be surprising given the different nature of the experiments, with TraDIS being in essence a very large competitive index experiment in which small differences in fitness can be sensitively identified, whereas the growth kinetics experiments we used measure the ability of mutants to grow in isolation.

Triclosan has become endemic in modern life and has been shown to impact microbes in the environment (Li et al. 2019). Our work suggests diverse residual concentrations of triclosan may result in very different selective pressures, and these findings may help inform the risk of the selection of unintended cross-resistance to other drugs. There have been multiple reports suggesting links between triclosan and antibiotic resistance. A recent paper described a link between triclosan exposure and induction of levels of ppGpp, which in turn result in up to 10,000-fold levels of protection against bactericidal antibiotics (Westfall et al. 2019). We saw no signal for *spoT* or *relA* (genes implicated in increasing ppGpp levels in that study). It may be that increasing ppGpp levels is not itself protective against triclosan or that “hard-wired” mutations are not needed to allow a fully protective response to occur after exposure, and hence, we observed no altered pattern of inserts at these loci in the presence of triclosan.

Multidrug efflux pumps are known to influence triclosan sensitivity, and the structural components of the pumps themselves were not protected by exposure to subinhibitory concentrations of triclosan although derepression of *marA*, which controls efflux expression, was selected. At concentrations above the MIC, the AcrAB efflux system becomes critically important, with mutants identified that will lead to pump overexpression through induction of SoxS or inactivation of *acrR* and protection of *acrAB* itself. Similarly, inactivation of *mprA* with consequent augmented transcription of the *emrAB* efflux system was also observed when exposed to concentrations of triclosan above the MIC. Selection of mutants that overexpress these regulators is a well-known route to multidrug resistance and suggests exposure to triclosan concentrations above the MIC is likely to select for antibiotic cross-resistance.

One currently unclear aspect of triclosan's activity relates to how it enters the cell: Although it is well established that triclosan crosses the outer membrane through porins, there is no current evidence about how triclosan crosses the inner membrane. Recent debate has highlighted the importance of transporters in the uptake of many small molecules rather than lipid diffusion (Kell and Oliver 2014). We identified two uptake systems (for ribose and potassium) whose inactivation results in increased resistance to triclosan. Specific mutants in these systems showed an increased ability to grow in the presence of triclosan (Fig. 3), suggesting they may have a role in drug import. Further work will be needed to substantiate the role of these channels in triclosan uptake.

The mechanism by which triclosan exerts a bactericidal effect is also currently elusive. We observed a pattern of genes involved in the protection of mRNA and protein stability, as well as translation initiation as being involved in triclosan sensitivity when exposed to high concentrations of the drug (Figs. 4, 6). These observations suggest the cells’ central information processing mechanism is compromised by high concentrations of triclosan. It remains to be seen if there is one or multiple secondary targets for triclosan that confer the bactericidal effect at high concentrations.

In this work, we show an improvement in transposon-based functional genomics, reveal multiple new aspects of the mechanisms of action and resistance of the canonical fatty acid biosynthesis inhibitor triclosan, and use this information to assess how differential exposure conditions impose distinct selective pressures. The use of an inducible promoter allows the impacts of overexpression and repression of all genes on phenotypes to be assayed efficiently. The ability to compare different conditions rapidly and to assay essential genes opens the door to high-throughput, genome-scale examination of bacterial responses to various stressors. This approach can be applied to any question in which the evaluation of bacterial fitness is important.

## Methods

### TraDIS-Xpress: a refined approach allowing essential genes to be assayed for roles in conditional survival

Although the original TraDIS protocol has proved to be hugely powerful in assaying the role of the majority of genes of any given bacteria in surviving any given stress, essential genes cannot be assayed owing to the lethality of any insertions within them. To allow essential genes to be assayed for roles in any given stress, we included the use of an inducible, outward facing promoter into the transposon cassette. This allows controlled induction of transcription from the transposon insertion site: Inserts immediately upstream of or downstream from an essential gene will either up- or down-regulate the gene. Changes in the abundance of these inserts can therefore be assayed by our new TraDIS-Xpress protocol. To enable this approach, we included an outward-oriented IPTG-inducible *tac* promoter at the end of the kanamycin cassette, which allows controlled induction of expression with IPTG in *E. coli* (for controlled expression from the *tac* promoter in the transposon cassette as assayed using a beta-galactosidase assay, see Supplemental Fig. S6). We used *E. coli* strain BW25113 as a model organism for these experiments as *E. coli* has been well studied for triclosan mechanisms of action and resistance, is compatible with the use of the *tac* promoter for conditional changes in gene expression, and is the parent strain for the defined Keio-mutant library, allowing access to independent inactivation mutants to validate TraDIS predictions. Supplemental Table 3 shows all strains and vectors used in this study.

### Preparation of transposomes

Transposon Tnp001 is a mini-Tn*5* transposon coding for kanamycin resistance (*aph*(*3*′)*-Ia*) that incorporates an outward oriented, IPTG-inducible *tac* promoter, 3′ to the kanamycin-resistance gene. Transposon DNA was amplified by PCR using oligonucleotides (primers are in Supplemental Table 4), and transposomes were prepared by mixing purified transposon DNA with EZ-Tn5 transposase according to the supplier's instructions (Lucigen). The mixture was incubated for 1 h at 37°C and then stored at −20°C before use.

### Preparation of a BW25113-mutant library

*E. coli* strain BW25113 was prepared for electroporation as previously described (Dower et al. 1988). Bacteria were grown in 2× YT broth to an optical density of 0.3 (measured at 600 nm). After harvesting, bacteria were washed three times in 10% glycerol before finally being suspended in 10% glycerol. Electrotransformations were performed using a Bio-Rad “gene pulser II” electroporator set to 2400 V and 25 µF, coupled to a “pulse controller II” set to 200 Ω and using 2-mm electrode gap cuvettes. Immediately after the electric pulse, cells were suspended in 1 mL of super optimal broth with catabolite repression (S.O.C. medium; NEB) and incubated for 1 h at 37°C. The cell suspension was then spread on LB agar supplemented with kanamycin at 30 mg/L and incubated overnight at 37°C. Resulting colonies were harvested and pooled together in 15% glycerol in LB broth for storage at −70°C.

#### Triclosan exposure conditions

To determine the exposure conditions for experiments, the minimum concentration of triclosan needed to inhibit growth of BW25113 (MIC) was determined using a twofold dilution method in LB broth, the same growth medium that was used to perform the TraDIS-Xpress experiments. In addition, viable counts of BW25113 were performed to determine bactericidal/bacteriostatic impacts by preparing duplicate twofold serial dilutions of triclosan from 4 to 0.008 mg/L in LB broth, inoculated with ∼10^6^ cfu/mL and incubated at 37°C. Cell numbers at 0 h were obtained from a 10 µL sample, removed immediately from each culture, and serially diluted 10-fold to a dilution of 10^−7^ in a 96-well plate. Five-microliter volumes of the dilutions were then spotted onto LB-agar, and the spots allowed to dry before incubation overnight at 37°C. After the LB broth cultures plus triclosan dilutions had been incubated for 24 h at 37°C, a second 10-µL sample was removed from each and treated similarly to obtain viable counts after 24 h in the presence of triclosan.

For TraDIS-Xpress experiments, approximately 10^7^ mutants from the transposon mutant library pool were grown in LB broth supplemented with a range of concentrations of triclosan in doubling increments ranging from 0.008 mg/L to 1 mg/L, representing a 125-fold range of concentrations. Experiments were also completed in the absence of IPTG or in the presence of either 0.2 or 1 mM IPTG to induce transcription from the transposon outward oriented promoter. Control experiments were also performed in the presence of two ethanol concentrations (ethanol was used to dissolve triclosan). All experiments were performed in duplicate. A total of 66 independent TraDIS-Xpress experiments were completed.

### Preparation of customized sequencing library

The pooled mutant mixtures were grown overnight in LB broth in the various conditions outlined above. DNA was extracted using a “quick-DNA” fungal/bacterial 96 kit extraction kit (Zymo Research). A Nextera DNA library preparation kit (Illumina) was used to prepare DNA fragments for nucleotide sequencing except that Tnp-i5 oligonucleotides were used instead of i5 index primers, and 28 PCR cycles were used (Hancock et al. 2017). The amplified DNA fragment mixture was selected for a size range of 300 bp–500 bp and sequenced on a NextSeq 500 sequencing machine using a NextSeq 500/550 high output v2 kit (75 cycles).

#### Bioinformatics

Sequence reads from FASTQ files generated from TraDIS sequencing were mapped to the BW25113 reference genome (CP009273) (Grenier et al. 2014) and analyzed using the Bio-TraDIS (version 1.4.1) (Barquist et al. 2016) and AlbaTraDIS (version 0.0.5) (Page et al. 2019) toolkits. Bio-TraDIS was used to align sequence reads (using SMALT version 0.7.4) to the reference genome and to create insertion plots. To identify gene essentiality for the different conditions, genes that have very few or no insertions are identified. Within the Bio-TraDIS toolkit (tradis_essentiality.R), a threshold value for the number of insertions within essential genes is estimated using the observed bimodal distribution of insertion sites over genes when normalized for gene length (Langridge et al. 2009; Barquist et al. 2013). To compare the insertion patterns between different concentrations of triclosan (conditions) and in media only (control), the AlbaTraDIS pipeline was used. As the TraDIS-Xpress approach also provides data on the induction and repression of genes, we were interested in changes in frequency of insertion patterns, not only within genes but also in the regions flanking the gene. A preferential increase of insertions oriented toward the gene (5′ to 3′) in the upstream region is suggestive of modulation of expression. A preferential increase of insertions in the antisense direction of the coding region of a gene (3′ to 5′) in the downstream region is suggestive of inhibition of gene expression by production of an interfering RNA transcript. To identify these patterns, the AlbaTraDIS toolkit (albaTraDIS script using -a flag for use of annotation) calculates the number of “forward” and “reverse” insertions per gene and 198 bp upstream of and downstream from the gene (using annotation for CP009273) (Grenier et al. 2014) for all genes in all conditions and control. Then the number of sequence reads are modelled on a per-gene basis using a negative binomial distribution and an adapted exact test as implemented in edgeR (Robinson et al. 2010) followed by multiple testing correction (Benjamini and Hochberg 1995), which is used to identify significant differences (using default cut-offs of *q*-value ≤0.05, logFC ≥ 1, logCPM > 8) in insertion frequency between each condition and control. The result is a comprehensive comparison that predicts which genes are important under the test stress as well as an indication if a change in expression of a gene (either down- or up-regulated) has certain advantages for survival in the different concentrations of triclosan.

Mapping of insertion sites was visually inspected using “Artemis,” which was used to capture images of selected areas of the genomes of interest with insertions for figures. (Carver et al. 2012)

#### Validation experiments

A total of 78 mutants were selected from the Keio library of defined mutants to validate predictions from the TraDIS-Xpress data set (Baba et al. 2006). These were selected to include genes representing the major pathways identified as involved in triclosan sensitivity, as well as a set of six randomly selected genes with no predicted impact on triclosan sensitivity. The Keio library contains two independent insertional inactivated mutants for each gene, and both were used for each of the 78 targets. Two triclosan-resistant mutants were also included: a BW25113 mutant selected after exposure to high level triclosan (as in Webber et al. 2008b) and L702, a previously characterized highly triclosan-resistant mutant of *Salmonella typhimurium* (Webber et al. 2008b). Both carried substitutions within FabI (Gly93Val).

For each mutant, the MIC of triclosan was determined as previously detailed. In addition, the ability of all mutants to grow in the presence of three concentrations of triclosan (0.015, 0.125, and 0.5 mg/L) were determined over 24 h and compared with drug-free controls to allow for generic growth impacts to be identified. Experiments were run in 96-well format in 100 µL of LB broth inoculated with ∼1000 cfu. Growth was assessed by measuring absorbance of cultures at 600 nm every 60 min in a BMG FLUOstar Omega plate reader. Each experiment was duplicated, giving four data sets for each gene (two for each mutant allele; each repeated independently). BW25113 was included in all experiments, giving a total of 34 separate measurements for growth of the parent in each condition.

#### Estimation of fitness from growth curve data

Fitness of each strain was estimated by the ratio of the incremental AUC (iAUC) at 0.125 mg/L triclosan to the average of the iAUC at 0 mg/L and 0.015 mg/L triclosan. This ratio was calculated for each replication of each strain on each plate, leading to each growth curve at 0.125 mg/L; hence, the fitness of each strain at 0.125 mg/L triclosan is summarized by a value between zero and one. These ratios were then modelled by a generalized linear mixed model, using the “beta” distributional family with a logistic link, estimated using the brms (Bürkner 2018) package in R (R Core Team 2019), which provides an interface to the Stan platform for Bayesian inference (Carpenter et al. 2017). Zeros were avoided by adding the smallest nonzero ratio across the whole data set to every other value. A fixed effect with five levels was included for the group to which each genotype belonged (TraDIS-indicated genes, importers, controls, BW25113, *Salmonella*-positive controls) with separate crossed groups effects for odd and even mutations nested within genes, plates, and an individual-level group effect to account for overdispersion. Four chains with 5000 warm-up and 5000 sampling iterations were used. Default priors were used throughout. The fitness of each mutant compared with BW25113 was then described by sampling from the posterior distribution of the effect of each mutant minus the effect of BW25113. Coefficients correspond to the estimated effect of each mutation on the ratio of growth in 0.125 mg/L triclosan to growth under no triclosan, on the logistic (log-odds) scale.

### Examining the impacts of directed alteration of gene expression on triclosan sensitivity

To confirm predicted impacts of altering expression of target genes, a selection of genes of interest were cloned into the pBAD30 vector under control of an arabinose-inducible promoter using EcoRI and Xbal restriction sites. These constructs were then tested for their ability to grow in the presence of different concentrations of triclosan on agar and in broth, both in a range of arabinose concentrations to induce expression of the cloned genes.

## Data access

All sequence data generated in this study have been submitted to the EBI European Nucleotide Archive (https://www.ebi.ac.uk/ena/) under project accession number PRJEB29311, within which are control samples (ERR2854363–ERR2854366) and treated samples (ERR2854367–ERR2854382). Supplemental Table 5 details each accession and corresponding treatment. Growth kinetic data are provided as Supplemental Data.

## Competing interest statement

The authors declare no competing interests.

## Supplementary Material

Supplemental Material
