# Supplementary material for: TraDIS-Xpress: a high-resolution whole-genome assay identifies novel mechanisms of triclosan action and resistance
Source: Genome Res. 2020 Feb;30(2):239–49. doi: 10.1101/gr.254391.119 (PMC7050523; doi:10.1101/gr.254391.119)
Supplement: Supplemental Material [file supp_30_2_239__index.html]

TraDIS-Xpress: a high-resolution whole-genome assay identifies novel mechanisms of triclosan action and resistance — TraDIS-Xpress: a high-resolution whole-genome assay identifies novel mechanisms of triclosan action and resistance — Supplemental Material 

# TraDIS-Xpress: a high-resolution whole-genome assay identifies novel mechanisms of triclosan action and resistance

## Supplemental Material

- Supplemental\_Material.docx
- Supplement\_data.xlsx
