# Supplementary material for: TraDIS-Xpress: a high-resolution whole-genome assay identifies novel mechanisms of triclosan action and resistance
Source: Genome Res. 2020 Feb;30(2):239–49. doi: 10.1101/gr.254391.119 (PMC7050523; doi:10.1101/gr.254391.119)
Supplement: Supplemental Material [file supp_gr.254391.119_Supplemental_Material_.docx]

**Supplementary material**

**Table of contents**

***Supplementary figures***

Supplementary Figure S1. Overview of the TraDIS-Xpress workflow

Supplementary Figure S2. Density of the transposon library, coverage across the genome and reproducibility between replicates.

Supplementary Figure S3. Impact of use of inducible promoter on numbers of genes identified.

Supplementary Figure S4. Demonstration of inducible expression from the *tac* promoter in the transposon cassette

Supplementary Figure S5. Comparison of fitness predictions from AlbaTraDIS with growth data in the presence of triclosan.

Supplementary Figure S6. Demonstration of inducible expression from the *tac* promoter in the transposon cassette.

***Supplementary tables***

Supplementary table 1. List of loci identified as significant after exposure to different triclosan concentrations

Supplementary table 2. Approximate costs and time requirements to make a library of 500,000 mutants using the original and new methods.

Supplementary table 3. Strains and vectors

Supplementary table 4. Primer sequences

Supplementary table 5. Accession numbers of sequence datasets

**Supplementary figure S1. Overview of the TraDIS-Xpress workflow**

**
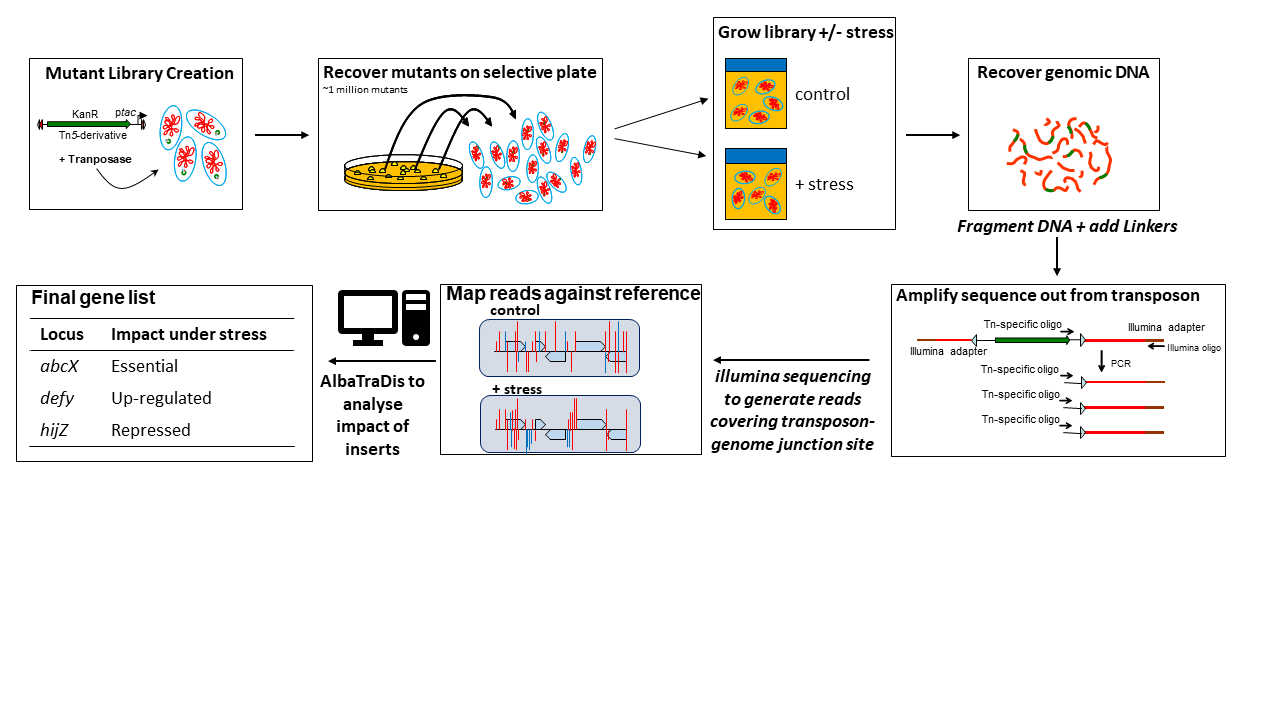
**

**Figure S1.** Illustration of the steps in generation of mutant library, growth under stress and analysis of insertion sites used in TraDIS-Xpress.

**Supplementary Figure S2. Density of the transposon library, coverage across the genome and reproducibility between replicates.**

**
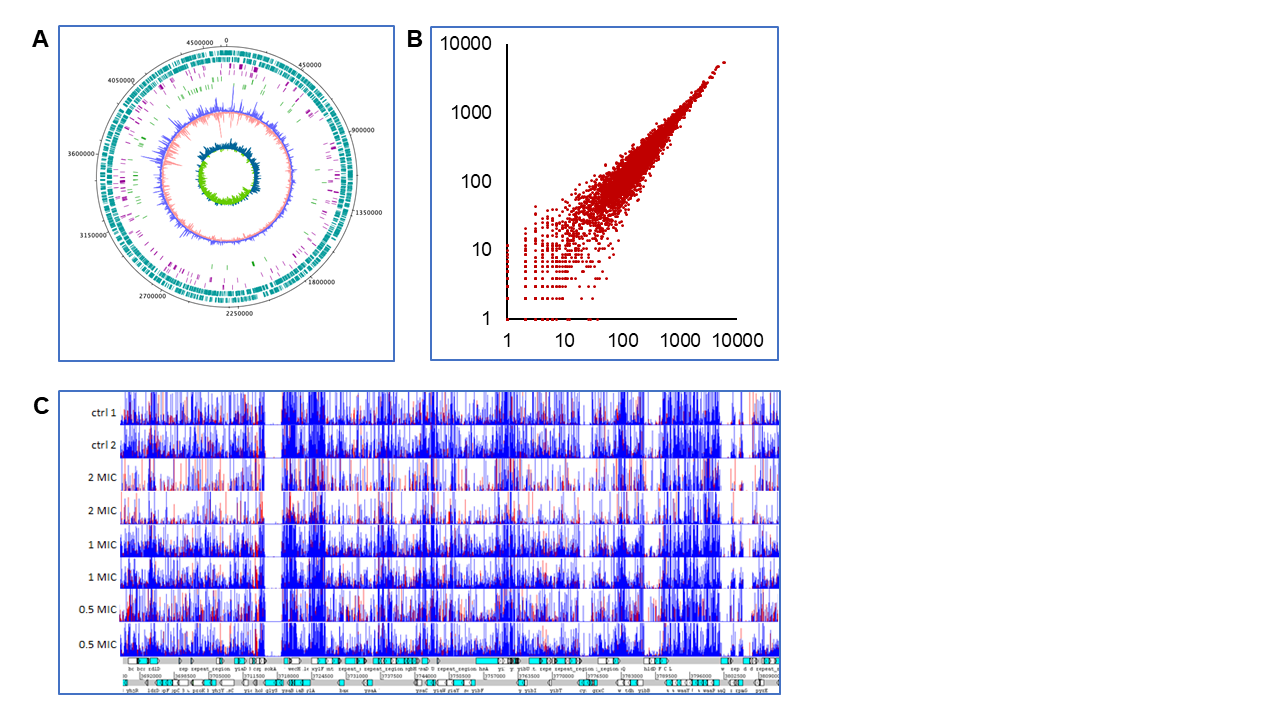
**

**Figure S2**. **Panel A** shows density of inserts across the genome of BW25113. From outside to in: Track 1, forward genes in BW25113; Track 2, reverse genes in BW25113; Track 3, essential genes in BW25113 (‘forward strand’); Track 4, essential genes in BW25113 (‘reverse strand’); Track 5, positions of genes showing conditional role in triclosan survival (‘forward’ strand); Track 6, positions of genes showing conditional role in triclosan survival (‘reverse’ strand); Track 7, ‘forward’ insertions in control library grown in LB 0 m*M* IPTG; Track 8, ‘reverse’ insertions in control library grown in LB 0 m*M* IPTG; Track 9, GC content. Insertion density around the origin is higher due to multiple copies present because the cells were growing exponentially prior to transposon mutagenesis. **Panel B** shows a scatter plot comparing numbers of inserts mapped against each gene isolated from two replicate libraries across the genome (points represent genes), the correlation coefficient gives an r value of 0.987 showing the consistency between replicates.  **Panel C** shows a 118 kb region of the *E. coli* BW25113 genome with mapped insertion sites to demonstrate the reproducibility of the data between biological replicates. A genetic map of the region is shown along the bottom. Above this, in each of the 8 horizontal windows, vertical lines show the location of transposon insertion sites, and the height of each bar reflects the number of transposon-directed sequence reads that mapped to each site. Red bars indicate insertions are orientated in line with the forward strand and blue bars (plotted over red) orientated in line with the reverse strand. Data from separate cultures are shown for each of four exposure conditions (no triclosan controls, 2× MIC, 1× MIC and 0.5× MIC of triclosan).

**Supplementary Figure S3. Impact of use of inducible promoter on numbers of genes identified.**

**Figure S3.** The number of loci identified as significant by AlbaTraDIS at each triclosan exposure condition. Blue bars show number of loci identified as being common to uninduced and induced conditions and red bars those only identified in the presence of induction. An average of 21% additional targets were identified in the presence of the inducer.

**Supplementary Figure S4. Growth of validation mutants compared against BW25113 in the presence of triclosan.**

**Figure S4.** Estimated average growth capacity (based on AUC of growth curves) of mutants in the presence of 0.125 mg/L of triclosan compared to growth of the same mutant grown without triclosan. Horizontal axis is the log-odds of the ratio between growth in 0.125mg/L triclosan and growth with no antimicrobial, compared to the same ratio in BW25113. Horizontal lines show 95% credible intervals for the effect of each estimate.

**Supplementary Figure S5. Comparison of fitness predictions from AlbaTraDIS with growth data in the presence of triclosan.**

**Figure S5.** Comparison of estimated effect on growth capacity of the presence of 0.125 mg/L of triclosan vs TraDIS-Xpress log fold change scores (data only shown from the 0.125 mg/L exposure)

**Supplementary Figure S6. Demonstration of inducible expression from the *tac* promoter in the transposon cassette**

**
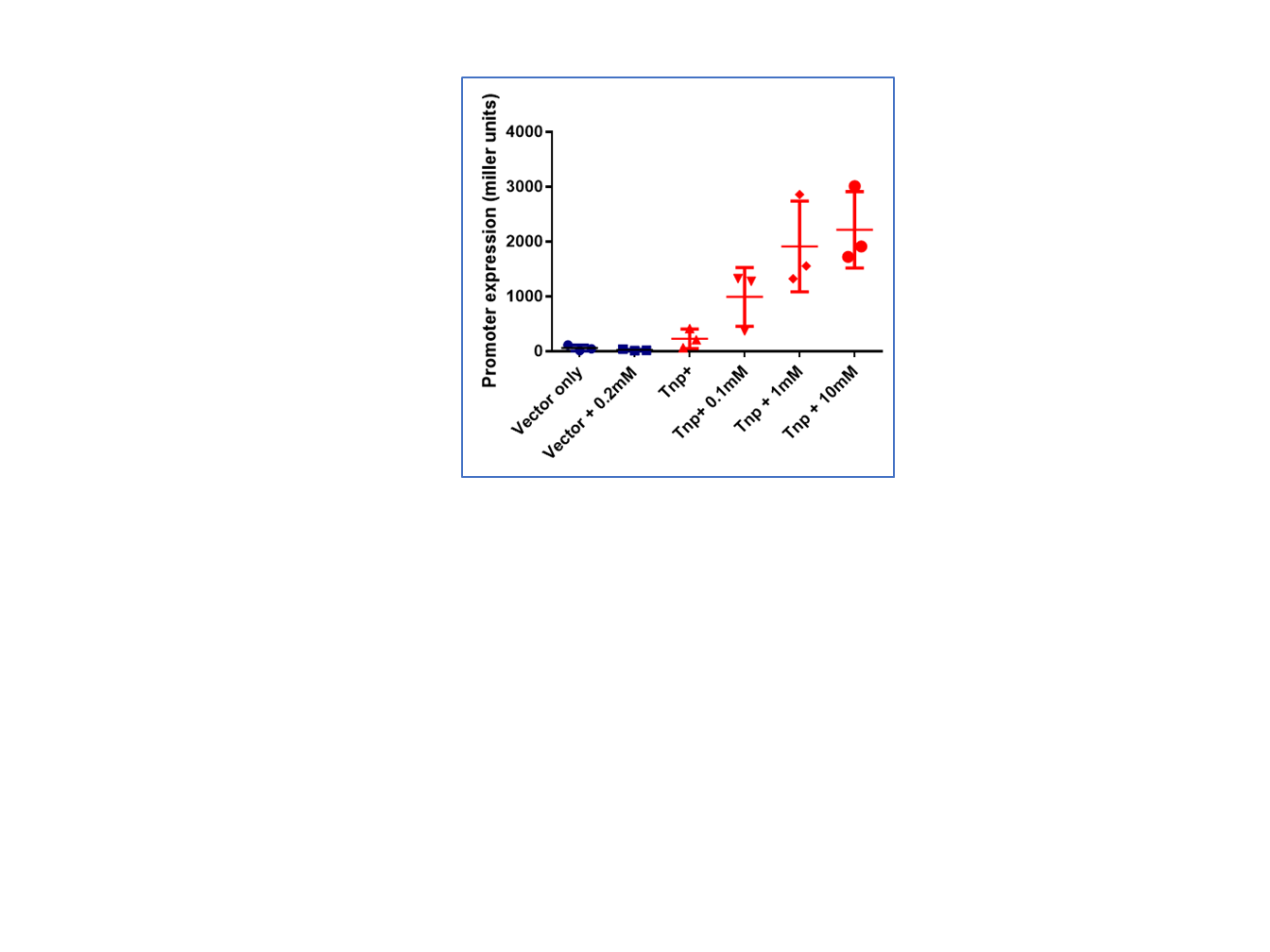
**

**Figure S6.** Data show miller units from β-galactosidase assays after different exposures to IPTG (concentrations marked), individual data points are shown along with mean (wide horizontal bars), and standard deviation (shorter horizontal bars).

**Supplementary tables**

**Supplementary table 1. List of loci identified as significant after exposure to different triclosan concentrations**

|  | **Triclosan concentration (mg/L)** | | | | | | | |
| --- | --- | --- | --- | --- | --- | --- | --- | --- |
| **Loci** | **0.8** | **0.15** | **0.3** | **0.6** | **0.12** | **0.25** | **0.5** | **1** |
| *aas* |  |  |  |  |  |  | 1.988 |  |
| *acrA* |  |  |  |  | 2.565 | 4.869 | 5.835 |  |
| *acrB* |  |  |  |  | -1.674 | -2.136 | -1.515 |  |
| *acrR* |  |  |  |  | 2.565 | 4.869 | 5.835 | 2.324 |
| *ampC* |  |  | 3.569 |  |  |  |  |  |
| *aroB* |  |  |  |  |  | 3.854 | 4.154 |  |
| *aroE* |  |  |  |  |  | 2.567 | 3.369 |  |
| *aroK* |  |  |  |  |  |  | 7.259 |  |
| *cpdB* |  |  |  | 1.225 |  |  |  |  |
| *creA* |  |  | 3.550 |  |  |  |  |  |
| *cyaA* |  |  |  |  |  | 2.358 | 1.528 |  |
| *cysQ* |  |  |  | 1.225 |  |  |  |  |
| *ecpA* | 1.291 | 1.142 | 1.125 | 1.375 |  |  |  |  |
| *ecpR* | -1.291 | -1.142 | -1.125 | -1.375 |  |  |  |  |
| *fabB* |  |  |  |  |  | 1.211 |  |  |
| *fabI* |  |  |  |  | 4.517 | 7.169 | 12.344 | 14.979 |
| *fabZ* |  |  |  |  |  |  | 8.367 |  |
| *fadD* |  |  |  |  |  |  | 3.245 |  |
| *fadL* |  |  |  |  |  | 1.228 | 1.837 |  |
| *fadR* |  |  |  |  |  | 3.451 | 3.685 |  |
| *fimA* |  |  |  |  |  | 1.580 |  |  |
| *fimB* |  |  |  |  |  |  | 1.681 |  |
| *fimC* |  |  |  |  |  |  | 1.313 |  |
| *fimD* |  |  |  |  |  |  | 1.325 | 1.765 |
| *fimE* |  |  |  |  |  | -1.696 | -2.943 | -2.589 |
| *fimI* | 1.756 | 1.395 |  |  |  |  | -1.646 |  |
| *folK* |  |  |  |  | 1.489 | 2.829 | 4.476 |  |
| *frdD* |  |  | 3.569 |  |  |  |  |  |
| *gadE* | -1.298 | -1.145 | -1.263 |  |  |  |  |  |
| *glf* | 1.433 | 1.135 | -1.229 | 1.231 |  |  |  |  |
| *gnd* | 1.189 |  | 1.144 |  |  |  |  |  |
| *hflC* |  |  |  |  |  |  | 1.274 |  |
| *hflK* |  |  |  |  |  |  | 1.485 |  |
| *infB* |  |  |  |  |  |  | 7.682 |  |
| *lon* |  |  |  |  |  | 0.000 | 0.000 |  |
| *lplT* |  |  |  |  |  |  | 1.988 |  |
| *lpxD* |  |  |  |  |  |  | 8.367 |  |
| *marC* |  |  | 7.267 |  |  |  |  |  |
| *marR* |  |  | 7.267 | 4.864 |  |  |  |  |
| *mcrC* | -1.223 |  | -1.447 | -1.196 |  |  |  |  |
| *metB* | -1.445 | -1.320 | -1.378 | -1.379 |  |  |  |  |
| *metL* | 1.445 | 1.320 | 1.378 | 1.379 |  |  |  |  |
| *mlaB* |  |  |  |  |  |  | 0.280 |  |
| *mlaC* |  |  |  |  |  | 0.165 | 0.268 |  |
| *mlaD* |  |  |  |  |  |  | 0.898 |  |
| *mlaE* |  |  |  |  |  |  | 0.169 | 0.534 |
| *mlaF* |  |  |  |  |  |  | 0.129 |  |
| *mnmC* |  |  |  |  |  | 1.211 |  |  |
| *mprA* |  |  |  |  |  | 3.284 | 3.599 |  |
| *mrcA* |  |  |  |  |  |  | -1.314 |  |
| *mscK* |  |  |  |  |  |  | 5.835 |  |
| *nusA* |  |  |  |  |  |  | 7.682 |  |
| *ompF* |  |  | 2.677 |  |  |  |  |  |
| *opgG* |  |  |  |  |  | 1.964 | 1.823 |  |
| *opgH* |  |  |  |  |  | 1.964 | 1.823 |  |
| *paoB* |  |  |  |  |  |  | 3.832 |  |
| *paoC* |  |  |  |  |  |  | 3.832 |  |
| *pcnB* |  |  |  |  | 1.489 | 2.829 | 4.476 |  |
| *phnP* |  |  |  |  |  |  | -5.235 | -5.997 |
| *ptsN* |  |  |  |  |  | 6.180 | 7.725 |  |
| *purH* |  | 1.128 | 1.640 | 1.534 |  |  |  |  |
| *purL* |  | 1.790 |  |  |  |  |  |  |
| *rbsB* | -1.613 |  | 2.349 |  |  |  |  |  |
| *rbsK* | 1.613 |  |  |  |  |  |  |  |
| *rcsC* |  |  |  |  |  |  | 1.140 |  |
| *rcsD* |  |  |  |  |  |  | 1.312 |  |
| *rfbC* |  |  | -1.822 |  |  |  |  |  |
| *rpoN* |  |  |  |  |  |  | 0.380 |  |
| *rsmB* |  |  |  |  |  | 0.260 | 0.000 |  |
| *sfmF* |  |  | -1.178 | -1.545 |  |  |  |  |
| *sfmZ* | -1.666 |  | -1.178 | -1.545 |  |  |  |  |
| *skp* |  |  |  |  |  | 3.378 | 4.478 |  |
| *soxR* |  |  |  |  |  |  | 3.859 |  |
| *soxS* |  |  |  |  |  |  | 3.859 |  |
| *tatC* | 1.969 | 1.959 | 1.858 | 1.678 |  | 1.312 |  |  |
| *trkA* |  |  |  |  |  | 3.938 | 4.716 |  |
| *trkG* | -1.144 |  |  |  |  |  |  |  |
| *trkH* |  |  |  |  |  | 1.478 | 1.883 |  |
| *tusA* |  |  |  |  |  |  | -6.491 | -6.332 |
| *ubiC* |  |  |  |  | 0.287 | 0.424 | 0.393 | 0.658 |
| *ubiD* |  |  |  |  |  |  | 0.000 |  |
| *ubiE* |  |  |  |  |  | 0.219 | 0.000 | 0.537 |
| *ubiF* |  |  |  |  | 0.311 | 0.000 | 0.000 | 0.328 |
| *uvrD* |  |  |  |  |  |  | 1.463 |  |
| *waaC* |  |  | -1.347 | -1.798 |  |  |  |  |
| *waaG* |  |  |  |  |  |  |  | 1.774 |
| *waaL* | -1.916 |  | -1.347 | -1.798 |  |  |  |  |
| *waaP* |  |  |  |  |  |  |  | 2.595 |
| *waaU* | -1.916 |  | -1.113 |  |  |  |  |  |
| *waaY* | -1.314 |  | -1.252 |  |  |  |  |  |
| *waaZ* | 1.314 |  | 1.252 |  |  |  |  |  |
| *wbbH* | -1.358 | -1.854 | -1.388 | -1.277 |  |  |  |  |
| *wbbI* |  |  | -1.719 | 1.277 |  |  |  |  |
| *wbbJ* |  |  | 1.719 |  |  |  |  |  |
| *wbbL* | -1.189 |  | -1.144 |  |  |  |  |  |
| *wzxB* | -1.433 | -1.135 | -1.314 | -1.231 |  |  |  |  |
| *xylA* |  |  |  | 1.117 |  |  |  |  |
| *yagH* | 1.666 | 1.387 |  |  |  |  |  |  |
| *yagL* | -1.265 | -1.346 | -1.179 |  |  |  |  |  |
| *ybcK* |  |  | -1.730 |  |  |  |  |  |
| *ycjD* |  |  |  |  | 4.517 | 7.169 | 12.344 | 14.979 |
| *ygeH* | -1.198 |  | -1.131 |  |  |  |  |  |
| *yhbJ* |  |  |  |  |  | 6.180 | 7.725 | 5.215 |
| *yhfL* |  |  | -1.225 |  |  |  |  |  |
| *yhiL* | -1.169 |  | -1.169 | -1.479 |  |  |  |  |
| *yhiS* | -1.440 | -1.384 | -1.579 | -1.335 |  |  |  |  |
| *yibA* | -1.168 | -1.134 | -1.327 | -1.142 |  |  |  |  |
| *yibB* |  |  | -1.132 |  |  |  |  |  |
| *yjbI* | -1.541 |  | -1.219 |  |  |  |  |  |
| *yjbM* | -1.737 | -1.155 | -1.374 | -1.926 |  |  |  |  |
| *yjcF* | -1.344 | -1.768 | -1.564 | -1.257 |  |  |  |  |
| *yjdP* |  |  |  |  |  |  | 5.235 | 5.997 |
| *yjgL* | -1.176 |  | -1.716 | -1.372 |  |  |  |  |
| *yjjQ* |  | 1.876 | 1.850 | 1.693 |  |  |  |  |
| *ykgA* |  |  | -1.172 |  |  |  |  |  |
| *ypjC* | -1.698 | -1.432 | -1.627 | -1.317 |  |  |  |  |
| *yrdB* |  |  |  |  |  | 2.567 | 3.369 |  |
| *zntA* |  |  |  |  |  |  | 6.491 | 6.332 |

Loci selected by adjusted p values of <0.05 are shown, values indicate log2 fold change values output by AlbaTraDIS. NB loci indicated are closest to insertion site – the impact of the insert may be predicted to be on nearby genes which will be referred to in the main text.

**Supplementary table 2. Approximate costs and time requirements to make a library of 500,000 mutants using the original and new methods.**

|  | **Original TraDIS** | | **TraDIS-Xpress** | |
| --- | --- | --- | --- | --- |
|  | Cost (£) | Time (h) | Cost (£) | Time (h) |
| Creation of mutants | 600 | 30 | 200 | 3 |
| DNA extraction per sample | 14 | 7 | 3 | 5 |
| Sequencing library prep | 70 | 8 | 60 | 4 |
| Sequencing | 31 | 48 | 3 | 12 |
| Analysis of the data (per single condition) | 0 | 4 | 0 | 1 |
| **Total** | **715** | **97** | **266** | **25** |

**Supplementary table 3. Strains and vectors**

|  | Strain or Plasmid | Description / Genotype | Reference |
| --- | --- | --- | --- |
| 1 | BW25113 | lacI^q^, rrnBT14, ∆lacZWJ16, hsdR514, ∆araBADAH33, ∆rhaBADLD78 | Baba et al 2006 |
| 2 | BW25113soxR::aph(3´)Ia | Wild type BW25113,soxR gene has been inactivated by aph(3´)Ia insertion | Baba et al 2006 |
| 3 | BW25113fadR::aph(3´)Ia | Wild type BW25113,fadR gene has been inactivated by aph(3´)Ia insertion | Baba et al 2006 |
| 4 | BW25113frdD::aph(3´)Ia | Wild type BW25113,frdD gene has been inactivated by aph(3´)Ia insertion | Baba et al 2006 |
| 5 | BW25113nuoG::aph(3´)Ia | Wild type BW25113,nuoG gene has been inactivated by aph(3´)Ia insertion | Baba et al 2006 |
| 6 | BW25113rbsB::aph(3´)Ia | Wild type BW25113,rbsB gene has been inactivated by aph(3´)Ia insertion | Baba et al 2006 |
| 7 | BW25113ompF::aph(3´)Ia | Wild type BW25113,ompF gene has been inactivated by aph(3´)Ia insertion | Baba et al 2006 |
| 8 | BW25113fadL::aph(3´)Ia | Wild type BW25113,fadL gene has been inactivated by aph(3´)Ia insertion | Baba et al 2006 |
| 9 | BW25113lon::aph(3´)Ia | Wild type BW25113,lon gene has been inactivated by aph(3´)Ia insertion | Baba et al 2006 |
| 10 | BW25113prc::aph(3´)Ia | Wild type BW25113,prc gene has been inactivated by aph(3´)Ia insertion | Baba et al 2006 |
| 11 | BW25113mrcA::aph(3´)Ia | Wild type BW25113,mrcA gene has been inactivated by aph(3´)Ia insertion | Baba et al 2006 |
| 12 | BW25113yehK::aph(3´)Ia | Wild type BW25113,yehK gene has been inactivated by aph(3´)Ia insertion | Baba et al 2006 |
| 13 | BW25113ybfQ::aph(3´)Ia | Wild type BW25113,ybfQ gene has been inactivated by aph(3´)Ia insertion | Baba et al 2006 |
| 14 | BW25113truD::aph(3´)Ia | Wild type BW25113,truD gene has been inactivated by aph(3´)Ia insertion | Baba et al 2006 |
| 15 | BW25113mprA::aph(3´)Ia | Wild type BW25113,mprA gene has been inactivated by aph(3´)Ia insertion | Baba et al 2006 |
| 16 | BW25113ubiF::aph(3´)Ia | Wild type BW25113,ubiF gene has been inactivated by aph(3´)Ia insertion | Baba et al 2006 |
| 17 | BW25113ybeX::aph(3´)Ia | Wild type BW25113,ybeX gene has been inactivated by aph(3´)Ia insertion | Baba et al 2006 |
| 18 | BW25113rep::aph(3´)Ia | Wild type BW25113,rep gene has been inactivated by aph(3´)Ia insertion | Baba et al 2006 |
| 19 | BW25113fadD::aph(3´)Ia | Wild type BW25113,fadD gene has been inactivated by aph(3´)Ia insertion | Baba et al 2006 |
| 20 | BW25113ydjO::aph(3´)Ia | Wild type BW25113,ydjO gene has been inactivated by aph(3´)Ia insertion | Baba et al 2006 |
| 21 | BW25113yedN::aph(3´)Ia | Wild type BW25113,yedN gene has been inactivated by aph(3´)Ia insertion | Baba et al 2006 |
| 22 | BW25113yhbJ::aph(3´)Ia | Wild type BW25113,yhbJ gene has been inactivated by aph(3´)Ia insertion | Baba et al 2006 |
| 23 | BW25113yibA::aph(3´)Ia | Wild type BW25113,yibA gene has been inactivated by aph(3´)Ia insertion | Baba et al 2006 |
| 24 | BW25113yjcF::aph(3´)Ia | Wild type BW25113,yjcF gene has been inactivated by aph(3´)Ia insertion | Baba et al 2006 |
| 25 | BW25113yjbI::aph(3´)Ia | Wild type BW25113,yjbI gene has been inactivated by aph(3´)Ia insertion | Baba et al 2006 |
| 26 | BW25113yjbL::aph(3´)Ia | Wild type BW25113,yjbL gene has been inactivated by aph(3´)Ia insertion | Baba et al 2006 |
| 27 | BW25113yigF::aph(3´)Ia | Wild type BW25113,yigF gene has been inactivated by aph(3´)Ia insertion | Baba et al 2006 |
| 28 | BW25113acrB::aph(3´)Ia | Wild type BW25113,acrB gene has been inactivated by aph(3´)Ia insertion | Baba et al 2006 |
| 29 | BW25113aroE::aph(3´)Ia | Wild type BW25113,aroE gene has been inactivated by aph(3´)Ia insertion | Baba et al 2006 |
| 30 | BW25113aroD::aph(3´)Ia | Wild type BW25113,aroD gene has been inactivated by aph(3´)Ia insertion | Baba et al 2006 |
| 31 | BW25113aroC::aph(3´)Ia | Wild type BW25113,aroC gene has been inactivated by aph(3´)Ia insertion | Baba et al 2006 |
| 32 | BW25113fre::aph(3´)Ia | Wild type BW25113,fre gene has been inactivated by aph(3´)Ia insertion | Baba et al 2006 |
| 33 | BW25113sspA::aph(3´)Ia | Wild type BW25113,sspA gene has been inactivated by aph(3´)Ia insertion | Baba et al 2006 |
| 34 | BW25113minC::aph(3´)Ia | Wild type BW25113,minC gene has been inactivated by aph(3´)Ia insertion | Baba et al 2006 |
| 35 | BW25113gcvP::aph(3´)Ia | Wild type BW25113,gcvP gene has been inactivated by aph(3´)Ia insertion | Baba et al 2006 |
| 36 | BW25113treC::aph(3´)Ia | Wild type BW25113,treC gene has been inactivated by aph(3´)Ia insertion | Baba et al 2006 |
| 37 | BW25113galU::aph(3´)Ia | Wild type BW25113,galU gene has been inactivated by aph(3´)Ia insertion | Baba et al 2006 |
| 38 | BW25113carB::aph(3´)Ia | Wild type BW25113,carB gene has been inactivated by aph(3´)Ia insertion | Baba et al 2006 |
| 39 | BW25113purL::aph(3´)Ia | Wild type BW25113,purL gene has been inactivated by aph(3´)Ia insertion | Baba et al 2006 |
| 40 | BW25113creA::aph(3´)Ia | Wild type BW25113,creA gene has been inactivated by aph(3´)Ia insertion | Baba et al 2006 |
| 41 | BW25113cyaA::aph(3´)Ia | Wild type BW25113,cyaA gene has been inactivated by aph(3´)Ia insertion | Baba et al 2006 |
| 42 | BW25113sapD::aph(3´)Ia | Wild type BW25113,sapD gene has been inactivated by aph(3´)Ia insertion | Baba et al 2006 |
| 43 | BW25113psta::aph(3´)Ia | Wild type BW25113,psta gene has been inactivated by aph(3´)Ia insertion | Baba et al 2006 |
| 44 | BW25113ptsN::aph(3´)Ia | Wild type BW25113,ptsN gene has been inactivated by aph(3´)Ia insertion | Baba et al 2006 |
| 45 | BW25113trkA::aph(3´)Ia | Wild type BW25113,trkA gene has been inactivated by aph(3´)Ia insertion | Baba et al 2006 |
| 46 | BW25113rsmB::aph(3´)Ia | Wild type BW25113,rsmB gene has been inactivated by aph(3´)Ia insertion | Baba et al 2006 |
| 47 | BW25113tatC::aph(3´)Ia | Wild type BW25113,tatC gene has been inactivated by aph(3´)Ia insertion | Baba et al 2006 |
| 48 | BW25113NusB::aph(3´)Ia | Wild type BW25113,NusB gene has been inactivated by aph(3´)Ia insertion | Baba et al 2006 |
| 49 | BW25113greA::aph(3´)Ia | Wild type BW25113,greA gene has been inactivated by aph(3´)Ia insertion | Baba et al 2006 |
| 50 | BW25113metL::aph(3´)Ia | Wild type BW25113,metL gene has been inactivated by aph(3´)Ia insertion | Baba et al 2006 |
| 51 | BW25113yhiL::aph(3´)Ia | Wild type BW25113,yhiL gene has been inactivated by aph(3´)Ia insertion | Baba et al 2006 |
| 52 | BW25113wzzE::aph(3´)Ia | Wild type BW25113,wzzE gene has been inactivated by aph(3´)Ia insertion | Baba et al 2006 |
| 53 | BW25113fimI::aph(3´)Ia | Wild type BW25113,fimI gene has been inactivated by aph(3´)Ia insertion | Baba et al 2006 |
| 54 | BW25113trkH::aph(3´)Ia | Wild type BW25113,trkH gene has been inactivated by aph(3´)Ia insertion | Baba et al 2006 |
| 55 | BW25113dgoR::aph(3´)Ia | Wild type BW25113,dgoR gene has been inactivated by aph(3´)Ia insertion | Baba et al 2006 |
| 56 | BW25113ybcV::aph(3´)Ia | Wild type BW25113,ybcV gene has been inactivated by aph(3´)Ia insertion | Baba et al 2006 |
| 57 | BW25113marR::aph(3´)Ia | Wild type BW25113,marR gene has been inactivated by aph(3´)Ia insertion | Baba et al 2006 |
| 58 | BW25113ypjC::aph(3´)Ia | Wild type BW25113,ypjC gene has been inactivated by aph(3´)Ia insertion | Baba et al 2006 |
| 59 | BW25113yabP::aph(3´)Ia | Wild type BW25113,yabP gene has been inactivated by aph(3´)Ia insertion | Baba et al 2006 |
| 60 | BW25113ycjD::aph(3´)Ia | Wild type BW25113,ycjD gene has been inactivated by aph(3´)Ia insertion | Baba et al 2006 |
| 61 | BW25113ubiH::aph(3´)Ia | Wild type BW25113,ubiH gene has been inactivated by aph(3´)Ia insertion | Baba et al 2006 |
| 62 | BW25113glpR::aph(3´)Ia | Wild type BW25113,glpR gene has been inactivated by aph(3´)Ia insertion | Baba et al 2006 |
| 63 | BW25113ybbC::aph(3´)Ia | Wild type BW25113,ybbC gene has been inactivated by aph(3´)Ia insertion | Baba et al 2006 |
| 64 | BW25113gadE::aph(3´)Ia | Wild type BW25113,gadE gene has been inactivated by aph(3´)Ia insertion | Baba et al 2006 |
| 65 | BW25113ompX::aph(3´)Ia | Wild type BW25113,ompX gene has been inactivated by aph(3´)Ia insertion | Baba et al 2006 |
| 66 | BW25113purH::aph(3´)Ia | Wild type BW25113,purH gene has been inactivated by aph(3´)Ia insertion | Baba et al 2006 |
| 67 | BW25113acrR::aph(3´)Ia | Wild type BW25113,acrR gene has been inactivated by aph(3´)Ia insertion | Baba et al 2006 |
| 68 | BW25113kgtP::aph(3´)Ia | Wild type BW25113,kgtP gene has been inactivated by aph(3´)Ia insertion | Baba et al 2006 |
| 69 | BW25113napD::aph(3´)Ia | Wild type BW25113,napD gene has been inactivated by aph(3´)Ia insertion | Baba et al 2006 |
| 70 | BW25113pcnB::aph(3´)Ia | Wild type BW25113,pcnB gene has been inactivated by aph(3´)Ia insertion | Baba et al 2006 |
| 71 | BW25113aroK::aph(3´)Ia | Wild type BW25113,aroK gene has been inactivated by aph(3´)Ia insertion | Baba et al 2006 |
| 72 | BW25113ynfD::aph(3´)Ia | Wild type BW25113,ynfD gene has been inactivated by aph(3´)Ia insertion | Baba et al 2006 |
| 73 | BW25113zntA::aph(3´)Ia | Wild type BW25113,zntA gene has been inactivated by aph(3´)Ia insertion | Baba et al 2006 |
| 74 | BW25113yhip::aph(3´)Ia | Wild type BW25113,yhip gene has been inactivated by aph(3´)Ia insertion | Baba et al 2006 |
| 75 | BW25113phnP::aph(3´)Ia | Wild type BW25113,phnP gene has been inactivated by aph(3´)Ia insertion | Baba et al 2006 |
| 76 | BW25113uhpT::aph(3´)Ia | Wild type BW25113,uhpT gene has been inactivated by aph(3´)Ia insertion | Baba et al 2006 |
| 77 | BW25113cutC::aph(3´)Ia | Wild type BW25113,cutC gene has been inactivated by aph(3´)Ia insertion | Baba et al 2006 |
| 78 | BW25113cstA::aph(3´)Ia | Wild type BW25113,cstA gene has been inactivated by aph(3´)Ia insertion | Baba et al 2006 |
| 79 | BW25113yjjQ::aph(3´)Ia | Wild type BW25113,yjjQ gene has been inactivated by aph(3´)Ia insertion | Baba et al 2006 |
| 80 | BW25113yjgL::aph(3´)Ia | Wild type BW25113,yjgL gene has been inactivated by aph(3´)Ia insertion | Baba et al 2006 |
| 81 | BW25113glpT::aph(3´)Ia | Wild type BW25113,glpT gene has been inactivated by aph(3´)Ia insertion | Baba et al 2006 |
| 82 | BW25113mcrC::aph(3´)Ia | Wild type BW25113,mcrC gene has been inactivated by aph(3´)Ia insertion | Baba et al 2006 |
| 83 | pBAD30 | A derivative of pBAD vector series carrying restriction sites to clone the gene of interest under the control of the araBAD promoter and ampicillin resistance cassette. | Guzman et al 1995 |
| 84 | pBAD/fabI | A derivative of pBAD30 carrying fabI cloned using EcoRI-XbaI under the control of the araBAD promoter | This study |
| 85 | pBAD/infB | A derivative of pBAD30 carrying infB cloned using EcoRI-XbaI under the control of the araBAD promoter | This study |
| 86 | pBAD/marAB | A derivative of pBAD30 carrying marAB cloned using EcoRI-XbaI under the control of the araBAD promoter | This study |
| 87 | pBAD/fabZ | A derivative of pBAD30 carrying fabZ cloned using EcoRI-XbaI under the control of the araBAD promoter | This study |
| 88 | pBAD/fabA | A derivative of pBAD30 carrying fabA in reverse orientation cloned using EcoRI-XbaI under the control of the araBAD promoter | This study |
| 89 | pBAD/rpoN | A derivative of pBAD30 carrying part of ropN in reverse orientation cloned using EcoRI-XbaI under the control of the araBAD promoter | This study |
| 90 | pBAD/pstA | A derivative of pBAD30 carrying pstA in reverse orientation cloned using EcoRI-XbaI under the control of the araBAD promoter | This study |
| 91 | pBAD/lacA | A derivative of pBAD30 carrying lacA in reverse orientation cloned using EcoRI-XbaI under the control of the araBAD promoter | This study |

**Supplementary table 4. Primer sequences**

| Primer name | Sequence (5´ to 3´) |  |  |  | Use |
| --- | --- | --- | --- | --- | --- |
| Tnp001P-i5S502-4 | AATGATACGGCGACCACCGAGATCTACACCTCTCTATACACTCTTTCCCTACACGACGCTCTTCCGATCTCTGACCAGGCATGCCAGGGTTGAGATGTG |  |  |  | Anneals to transposon specific Sequence and used in sequencing library preparation |
| Tnp001P-i5S502-5 | AATGATACGGCGACCACCGAGATCTACACCTCTCTATACACTCTTTCCCTACACGACGCTCTTCCGATCTTGACATCAGGCATGCCAGGGTTGAGATGTG |  |  |  | Anneals to transposon specific Sequence and used in sequencing library preparation |
| Tnp001P-i5S502-6 | AATGATACGGCGACCACCGAGATCTACACCTCTCTATACACTCTTTCCCTACACGACGCTCTTCCGATCTGACTGAGCAGGCATGCCAGGGTTGAGATGTG |  |  |  | Anneals to transposon specific Sequence and used in sequencing library preparation |
| Tnp001P-i5S502-7 | AATGATACGGCGACCACCGAGATCTACACCTCTCTATACACTCTTTCCCTACACGACGCTCTTCCGATCTACTGTGTTCAGGCATGCCAGGGTTGAGATGTG |  |  |  | Anneals to transposon specific Sequence and used in sequencing library preparation |
| Tnp001P-i5S503-4 | AATGATACGGCGACCACCGAGATCTACACTATCCTCTACACTCTTTCCCTACACGACGCTCTTCCGATCTCTGACCAGGCATGCCAGGGTTGAGATGTG |  |  |  | Anneals to transposon specific Sequence and used in sequencing library preparation |
| Tnp001P-i5S503-5 | AATGATACGGCGACCACCGAGATCTACACTATCCTCTACACTCTTTCCCTACACGACGCTCTTCCGATCTTGACATCAGGCATGCCAGGGTTGAGATGTG |  |  |  | Anneals to transposon specific Sequence and used in sequencing library preparation |
| Tnp001P-i5S503-6 | AATGATACGGCGACCACCGAGATCTACACTATCCTCTACACTCTTTCCCTACACGACGCTCTTCCGATCTGACTGAGCAGGCATGCCAGGGTTGAGATGTG |  |  |  | Anneals to transposon specific Sequence and used in sequencing library preparation |
| Tnp001P-i5S503-7 | AATGATACGGCGACCACCGAGATCTACACTATCCTCTACACTCTTTCCCTACACGACGCTCTTCCGATCTACTGTGTTCAGGCATGCCAGGGTTGAGATGTG |  |  |  | Anneals to transposon specific Sequence and used in sequencing library preparation |
| Tnp001P-i5S505-4 | AATGATACGGCGACCACCGAGATCTACACGTAAGGAGACACTCTTTCCCTACACGACGCTCTTCCGATCTCTGACCAGGCATGCCAGGGTTGAGATGTG |  |  |  | Anneals to transposon specific Sequence and used in sequencing library preparation |
| Tnp001P-i5S505-5 | AATGATACGGCGACCACCGAGATCTACACGTAAGGAGACACTCTTTCCCTACACGACGCTCTTCCGATCTTGACATCAGGCATGCCAGGGTTGAGATGTG |  |  |  | Anneals to transposon specific Sequence and used in sequencing library preparation |
| Tnp001P-i5S505-6 | AATGATACGGCGACCACCGAGATCTACACGTAAGGAGACACTCTTTCCCTACACGACGCTCTTCCGATCTGACTGAGCAGGCATGCCAGGGTTGAGATGTG |  |  |  | Anneals to transposon specific Sequence and used in sequencing library preparation |
| Tnp001P-i5S505-7 | AATGATACGGCGACCACCGAGATCTACACGTAAGGAGACACTCTTTCCCTACACGACGCTCTTCCGATCTACTGTGTTCAGGCATGCCAGGGTTGAGATGTG |  |  |  | Anneals to transposon specific Sequence and used in sequencing library preparation |
| i7N701 | CAAGCAGAAGACGGCATACGAGATTCGCCTTAGTCTCGTGGGCTCGG |  |  |  | Standard Illumina indices for sequencing library preparation |
| i7N702 | CAAGCAGAAGACGGCATACGAGATCTAGTACGGTCTCGTGGGCTCGG |  |  |  | Standard Illumina indices for sequencing library preparation |
| i7N703 | CAAGCAGAAGACGGCATACGAGATTTCTGCCTGTCTCGTGGGCTCGG |  |  |  | Standard Illumina indices for sequencing library preparation |
| i7N704 | CAAGCAGAAGACGGCATACGAGATGCTCAGGAGTCTCGTGGGCTCGG |  |  |  | Standard Illumina indices for sequencing library preparation |
| i7N705 | CAAGCAGAAGACGGCATACGAGATAGGAGTCCGTCTCGTGGGCTCGG |  |  |  | Standard Illumina indices for sequencing library preparation |
| i7N706 | CAAGCAGAAGACGGCATACGAGATCATGCCTAGTCTCGTGGGCTCGG |  |  |  | Standard Illumina indices for sequencing library preparation |
| i7N707 | CAAGCAGAAGACGGCATACGAGATGTAGAGAGGTCTCGTGGGCTCGG |  |  |  | Standard Illumina indices for sequencing library preparation |
| i7N710 | CAAGCAGAAGACGGCATACGAGATCAGCCTCGGTCTCGTGGGCTCGG |  |  |  | Standard Illumina indices for sequencing library preparation |
| i7N711 | CAAGCAGAAGACGGCATACGAGATTGCCTCTTGTCTCGTGGGCTCGG |  |  |  | Standard Illumina indices for sequencing library preparation |
| i7N712 | CAAGCAGAAGACGGCATACGAGATTCCTCTACGTCTCGTGGGCTCGG |  |  |  | Standard Illumina indices for sequencing library preparation |
| i7N714 | CAAGCAGAAGACGGCATACGAGATTCATGAGCGTCTCGTGGGCTCGG |  |  |  | Standard Illumina indices for sequencing library preparation |
| i7N715 | CAAGCAGAAGACGGCATACGAGATCCTGAGATGTCTCGTGGGCTCGG |  |  |  | Standard Illumina indices for sequencing library preparation |
| Tnp100 | CTGTCTCTTATACACATCT |  |  |  | Mosaic end of transposon and used for transposon amplification |
| D10520 | CCCTGCGGTGCCCCTCAAG |  |  |  | Anneals upstream of MCS in pRW50 vector and used for sequencing |
| D78264 | GGCTGTAATGTTCTGGCATTTGGTCAGC |  |  |  | Anneals downstream of MCS in pRW50 vector and used for sequencing |
| Tnp-seq 100-Fw | GCATTTTATCCGTACTCCTGATGATGCATG |  |  |  | Anneals Kanamycin cassette in transposon construct |
| FabI.forward for pbad30 | GGGGGGAATTCATCGCCTGATTTTCAGGCACAACAAG |  |  |  | used for fabI amplification |
| fabI.reverse. for pbad30 | GGGGGTCTAGAGATTATTTCAGTTCGAGTTCGTTCATTGC |  |  |  | used for fabI amplification |
| pstA.for.k.down pbad30 | GGCCGGAATTCAACAATATCAACCGTGTTTATTCTTCG |  |  |  | used for pstA amplification |
| pstA.rev.k.down pbad30 | GGCCGTCTAGAGCTATGGTTGAAATGCAAACCACTG |  |  |  | used for pstA amplification |
| rpoN.for.k.down pbad30 | GGCCGGAATTCGTCAAACGAGTTGTTTACGCTGG |  |  |  | used for rpoN amplification |
| rpoN.rev.kdown pbad30 | GGCCGTCTAGAAAGCAAGGTTTGCAACTCAGGC |  |  |  | used for rpoN amplification |
| infBfor pbad30 | GGCCGAATTCGTAATATTTGCTGGTTCGGTGACGAAG |  |  |  | used for infB amplification |
| infB rev pbad30 | GGGGTCTAGACTAAAAACCTTAAGCAATGGTACGTTGGATC |  |  |  | used for infB amplification |
| fabZ for pbad30 | GGCCGAATTCCTTTACGGCCTGTCTCATTCTTACGATTGCG |  |  |  | used for fabZ amplification |
| fabZ.rev pbad30 | GGCCGTCTAGAATCAGGCCTCCCGGCTACGAGC |  |  |  | used for fabZ amplification |
| lacA.for.k.downpbad30 | GGCCGGAATTCTTTAAACTGACGATTCAACTTTATAATCTTTGAAATAATAG |  |  |  | used for lacA amplification |
| lacA.rev.k.downpbad30 | GGCCGTCTAGAACATGCCAATGACCGAAAGAATAAGAGC |  |  |  | used for lacA amplification |
| fabA.forward.k.downpbad30 | GGCCGGAATTCTCAAGAAGGCAGACGTATCCTGGAAC |  |  |  | used for fabA amplification |
| fabA.rev.k.downpbad30 | GGCCGTCTAGAGTAGATAAACGCGAATCCTATACAAAAGAAGAC |  |  |  | used for fabA amplification |
| marAB for pbad30 | GGCCGAATTCGACGAAGTGGCAACACTTGAGTATTTGCTTAAGAAAGTC |  |  |  | used for marAB amplification |
| marAB.rev pbad30 | GGCCGTCTAGACTACATAGCGTGTTGATTATAATAGGG |  |  |  | used for marAB amplification |

**Supplementary table 5. Accession numbers of sequence data**

| **Triclosan (**mg/L) | **Accession** | |
| --- | --- | --- |
|  | Replicate 1 | Replicate 2 |
| 0.008 | ERR2854367 | ERR2854368 |
| 0.015 | ERR2854369 | ERR2854370 |
| 0.03 | ERR2854371 | ERR2854372 |
| 0.06 | ERR2854373 | ERR2854374 |
| 0.125 | ERR2854375 | ERR2854376 |
| 0.25 | ERR2854377 | ERR2854378 |
| 0.5 | ERR2854379 | ERR2854380 |
| 1.0 | ERR2854381 | ERR2854382 |
| Control 1 | ERR2854363 | ERR2854364 |
| Control 2 | ERR2854365 | ERR2854366 |
